# ASPS Exhibits Anti‐Rheumatic Effects by Reprogramming Gut Microbiota and Increasing Serum *γ*‐Glutamylcysteine Level

**DOI:** 10.1002/advs.202205645

**Published:** 2022-11-23

**Authors:** Ang Liu, Min Zhang, Yanglin Wu, Chenhui Zhang, Qin Zhang, Xinlin Su, Xu Zhu, Weidong Shi, Jiangyun Liu, Yang Zhang, Cheng Huang, Zhaowei Yan, Jun Lin

**Affiliations:** ^1^ Department of Orthopaedics Suzhou Dushu Lake Hospital Dushu Lake Hospital Affiliated to Soochow University Medical Center of Soochow University Suzhou 215125 China; ^2^ Department of Orthopaedics The First Affiliated Hospital of Soochow University Suzhou 215006 China; ^3^ Department of Pharmacy The First Affiliated Hospital of Soochow University Suzhou 215006 China; ^4^ College of Pharmaceutical Sciences Soochow University Suzhou 215123 China; ^5^ Department of Orthopaedics Shanghai Tenth People's Hospital School of Medicine Tongji University Shanghai 200092 China; ^6^ School of Biology and Food Engineering Changshu Institute of Technology Changshu 215500 China; ^7^ Department of Orthopaedics China‐Japan Friendship Hospital Beijing 100029 China

**Keywords:** *γ*‐glutamylcysteine, *Acanthopanax senticosus* polysaccharide, gut microbiota, metabolomics, rheumatoid arthritis

## Abstract

Rheumatoid arthritis (RA) is an essential cause of labor loss and disability for people worldwide. *Acanthopanax senticosus* polysaccharide (ASPS) is one of the most important active components from *A. senticosus*, which exhibits various pharmacological activities such as antioxidation and immunomodulation. However, no studies have reported the application of ASPS in treating RA. This study aims to investigate the therapeutic effect of ASPS on RA and reveal its underlying mechanism. The potential therapeutic effect of ASPS against RA is initially verified in this study using the collagen‐induced arthritis model. Moreover, the protective benefits of ASPS are transmitted through the fecal microbiota and blocked by simultaneous antibiotic cocktail treatment, indicating that gut microbiota may be correlated with ASPS. The 16S rRNA sequencing using feces samples and untargeted UPLC‐MS metabolomics using serum samples further reveal that ASPS reprograms the arthritic progression triggered dysbiosis, enhances the expression of *γ*‐glutamylcysteine (GGC) synthetase, and enriches the serum concentration of GGC. Furthermore, metabolites GGC is found to be able to effectively interrupt NLRP3 inflammasome activation via inhibiting ASC nucleation and therefore attenuate inflammatory arthritis. Taken together, this work highlights ASPS's therapeutic potential against RA, which mainly exhibits its effects via modulating gut microbiota and regulating GGC production.

## Graphical abstract

*Acanthopanax senticosus* polysaccharide (ASPS) exhibits anti‐rheumatic effects by reprogramming gut microbiota and increasing serum *γ*‐glutamylcysteine (GGC) level. ASPS reprograms gut microbiota and raises serum GGC concentration. Enrichment of GGC following the change of microbiota activates suppress arthritis via inhibiting NLRP3 inflammasome activation.

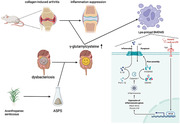

## 1 Introduction

Rheumatoid arthritis (RA) is characterized by synovitis, pannus formation, and erosive joint damage as the primary clinical manifestations, impacting almost 1–2% of the worldwide population.^[^ ^1^, ^2^ ^]^ RA is also characterized by the over‐activation, differentiation, and proliferation of lymphocytes.^[^ ^3^ ^]^ The disability rates of RA patients are 18.6%, 43.5%, 48.1%, and 61.3% for 1–5, 5–10, 10–15, and ≥15 years, respectively, and the disability rate increases with the duration of the disease. Despite recent advances in understanding its pathogenesis, with many genetic susceptibility risk alleles identified, the etiology of RA remains elusive.^[^ ^4^, ^5^, ^6^, ^7^ ^]^ There is increasing evidence that the gut‐immune axis exerts a vital role in regulating RA.^[^ ^8^, ^9^ ^]^ The abundance of a variety of pathogenic bacteria, including *Prevotella* and segmental filamentous bacteria, is related to the severity of RA.^[^ ^10^, ^11^ ^]^ The gut microbiota is considered one of the most relevant environmental factors influencing the development of RA.

*A. senticosus* is a widely used traditional Chinese medicine, mainly distributed in the Far East of Russia, South Korea, Japan, China, and other Northeast Asian countries.^[^ ^12^ ^]^ Although the stem and root of *A. senticosus* have traditionally been used to treat RA,^[^ ^13^, ^14^, ^15^ ^]^ the underlying mechanism remains unclear. *A. senticosus* polysaccharide (ASPS), one of the most important active components from *A. senticosus*, which exhibited various pharmacological activities such as antioxidant, antidiabetic, and immunomodulatory activities.^[^ ^16^, ^17^, ^18^ ^]^ A few studies have focused on its interaction with the gut microbiota. ASPS has a significant protective effect on intestinal homeostasis in *Drosophila melanogaste*r.^[^ ^19^ ^]^ ASPS could also ameliorate sepsis in mice by inhibiting the NF‐*κ*B/MLCK pathway to reduce intestinal tight junction damage.^[^ ^20^ ^]^ However, whether ASPS plays its anti‐rheumatic benefits indirectly through the regulation of gut microbiota is still undercover. Considering that alterations in the gut microbiota and its metabolites have a close impact on the development of RA, we envisioned that gut microbiota would be an entry point to explore the underlying mechanism of the anti‐rheumatic benefits of ASPS. In this study, we first investigated whether the protective effects of ASPS against RA are correlated with gut microbiota. We then screened the key differential metabolites associated with ASPS treatment and clarified how ASPS regulates metabolite levels through gut microbiota. Soon after, we tested the hypothesis that *γ*‐glutamylcysteine (GGC) may be a key factor in the anti‐rheumatic benefits of ASPS and that GGC can alleviate the RA symptoms in CIA mice. Finally, it was also demonstrated that the anti‐rheumatic effects of GGC depend on inhibiting NLRP3 inflammasome activation.

## 2 Results

### 2.1 The Anti‐Rheumatic Benefits of ASPS Were Correlated with Gut Microbiota

It is well known that the gut microbiota is susceptible to antibiotics.^[^ ^21^, ^22^, ^23^, ^24^ ^]^ Antibiotic cocktail therapy is a widely used method to greatly reduce the number of gut microbiota.^[^ ^25^, ^26^, ^27^ ^]^ In order to confirm whether the gut flora could affect rheumatoid arthritis, we administered antibiotic cocktail to mice with collagen‐induced arthritis (CIA). As arthritis progressed, CIA mice's paw arthritis scores quickly increased (Figure S1C,E, Supporting Information). However, the increase in arthritis scores was much faster in CIA mice treated with the antibiotic cocktail (Figure S1A,C, Supporting Information). Both arthritis scores (*p* < 0.05, Figure S1C,D, Supporting Information) and thickness changes (*p* < 0.001, Figure S1E, Supporting Information) were significantly increased in antibiotic cocktail‐treated mice compared to CIA mice at day 51 after primary immunization. Micro‐CT analysis demonstrated severe bone erosion in the ankle and finger joints of CIA mice (Figure S1B, Supporting Information). Whereas antibiotic cocktail treatment effectively enhanced the bone erosion activity, showing decreased BV/TV (*p* < 0.001, Figure S1F, Supporting Information) and increased total porosity (*p* < 0.001, Figure S1G, Supporting Information). We also observed severe cartilage damage and pannus formation in ATBX mice (Figure S1H–K, Supporting Information). These results prove that the removal of gut microbiota could aggravate rheumatoid arthritis.

**Figure 1:**
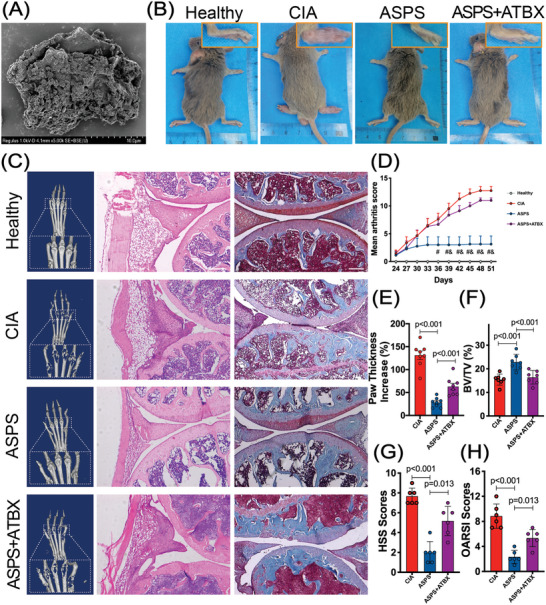
The gut microbiota mediated the anti‐rheumatic benefits of ASPS. 8 weeks old male DBA/1 mice were divided into four groups: Healthy, CIA (collagen‐induced arthritis model), ASPS (CIA mice treated with ASPS), and ASPS + ATBX (CIA mice simultaneously treated with ASPS and antibiotic cocktail). A) Representative scanning electron microscope image of ASPS (×5.0 K). B) Representative images of mice on the 51st day after primary immunization. C) Representative micro‐CT reconstruction image of the hind paws (left panel). Representative H&E stained (middle panel) and Safranin O stained (right panel) images of knee joints. Scale bar: 100 µm. D) Clinical arthritis score of the mice (*n* = 8). # indicates pASPS versus CIA < 0.05, & indicates pASPS versus ASPS + ATBX < 0.05. E) Hind paw thickness changes (*n* = 8). F) Quantitative analysis of bone volume over total volume ratio (BV/TV) (*n* = 7). G) Histological synovitis scores (HSS) of knee joints. (*n* = 6). H) Modified OARSI scores of knee joints. (*n* = 6). Statistical analyses were performed with Kruskal–Wallis one way analysis of variance on ranks followed by the post hoc Tukey's comparisons (D), or ANOVA followed by the post hoc Student–Newman–Keuls pairwise comparisons (E–H). Error bars indicate SEM.

The water‐soluble polysaccharide extracted from *A. senticosus* was purified by anion exchange and size exclusion chromatography, namely ASPS (**Figure** **1**A). ASPS showed a major peak with a molecular weight of 1.65 × 10^5^ Da and mainly contained d‐galactose with minor proportions of d‐glucose, l‐arabinose, d‐rhamnose, and d‐galacturonic acid (Figure S2, Supporting Information). To verify whether the gut microbiota mediated the anti‐rheumatic effects of ASPS, we simultaneously treated CIA mice with antibiotic cocktail and ASPS. As the disease progressed, the arthritis score of the paws increased rapidly in CIA mice (Figure 1B,D, and Figure S3A, Supporting Information). However, the arthritis score increased much more slowly in ASPS‐treated CIA mice. ASPS‐treated mice showed a significantly reduced arthritis score (*p* < 0.05, Figure 1D and Figure S3B, Supporting Information) and paw thickness change (*p* < 0.001, Figure 1E) compared with CIA mice on the 51st day after primary immunization. Interestingly, the antibiotic cocktail treatment significantly compromised the anti‐arthritis efficacy of ASPS. The arthritis score and paw thickness change increased dramatically by 252% and 122.92%, respectively (Figure 1E and Figure S3B, Supporting Information) in antibiotic cocktail‐treated mice. Micro‐CT analysis demonstrated severe bone erosion in the ankle and finger joints of CIA mice (Figure 1C). Whereas, ASPS treatment effectively inhibited the bone erosion activity, showing increased BV/TV (*p* < 0.001), BV (p<0.001),  BS (*p* = 0.002), MMI (*p* = 0.002), Tb.N (*p* = 0.001), and Tb.Th (*p* = 0.022), and total porosity (*p* < 0.001) (Figure 1F and Figure S3C–H, Supporting Information). Similarly, in the mice treated with antibiotic cocktail therapy, the inhibited bone erosion activity was also exacerbated. We can observe extensive pannus formation, inflammatory cell infiltration, proteoglycan loss, cartilage degradation, and destruction in CIA mice (Figure 1C), with the highest HSS and OARSI scores (Figure 1G,H). In addition, the level of IL‐1*β* and TNF‐*α* in serum were further measured by ELISA method, which also proved that ASPS treatment could significantly reduce the level of inflammatory cytokines IL‐1*β* and TNF‐*α* in serum (Figure S3I,J, Supporting Information). Compared with CIA mice, ASPS treatment also inhibited the expression of F4/80, caspase‐1, and IL‐1*β*, indicating the inhibition of inflammasome activation in vivo (Figure S4A–C, Supporting Information). ASPS treatment exhibited an effective ability of inhibiting cartilage damage, inflammatory cell infiltration, and pannus formation, while the antibiotic cocktail weakened this protective effect. Collectively, these results demonstrated that the anti‐rheumatic effects of ASPS were mediated by gut microbiota.

### 2.2 Fecal Microbiota Transplantation Mimicked the Anti‐Rheumatic Effect Similar to ASPS Treatment

**Figure 2:**
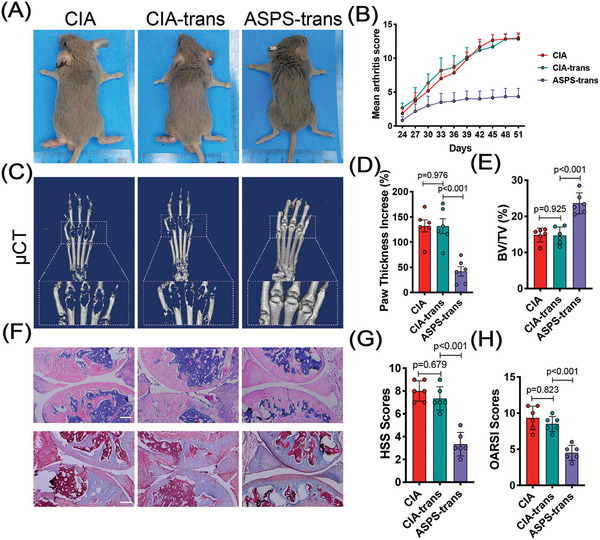
Fecal microbiota transplantation mimicked the anti‐rheumatic effect similar to ASPS treatment. 8‐week‐old male DBA/1 mice were divided into three groups: CIA (collagen‐induced arthritis model), CIA‐trans (fecal microbiome transplantation from CIA mice), and ASPS‐trans (fecal microbiome transplantation from ASPS treated mice). A) Representative view of mice on the 51st day after primary immunization (sacrifice). B) Clinical arthritis score of the mice (*n* = 6). C) Representative micro‐CT reconstruction image of the hind paws. D) Hind paw thickness changes (*n* = 6). E) Quantitative analysis of BV/TV (*n* = 6). F) Representative H&E stained (upper panel) and Safranin O stained (lower panel) images of knee joints. Scale bar: 100 µm. G) Histological synovitis scores (HSS) of knee joints (*n* = 6). H) Modified OARSI scores of knee joints (*n* = 6). Statistical analyses were performed with ANOVA followed by the post hoc Student–Newman–Keuls pairwise comparisons (D,E,G,H). Error bars indicate SEM.

Based on our previous study,^[^ ^27^ ^]^ antibiotic cocktail therapy can greatly reduce the number of gut microbes, and the number of microbes can substantially increase via microbial transplantation.^[^ ^28^ ^]^ To verify whether the gut microbiota mediates anti‐rheumatic benefits of ASPS, the fecal microbiota from ASPS‐treated mice was transmitted to antibiotic cocktail therapy treated CIA mice. As expected, fecal microbiota transplantation exhibited a similar reduction in arthritis symptoms compared to ASPS treatment. Reduced arthritis score (**Figure** **2**A,B) and paw thickness changes (Figure 2D), improved bone erosion (Figure 2C,E), cartilage damage (Figure 2F,H), and pannus formation (Figure 2F,G) all prove that fecal microbiota transplantation could mimic the anti‐rheumatic effect similar to ASPS treatment. In addition, the level of IL‐1*β* in serum was further measured by the ELISA method, which also proved that fecal microbiota transplantation could significantly reduce the level of inflammatory cytokines IL‐1*β* in serum (Figure S5D, Supporting Information). Compared with CIA‐trans mice, the expression of F4/80, caspase‐1, and IL‐1*β* in ASPS‐trans mice also decreased, indicating that the transfer could inhibit inflammasome activation (Figure S5A–C, Supporting Information).

### 2.3 ASPS Reprogramed Gut Microbiota

**Figure 3:**
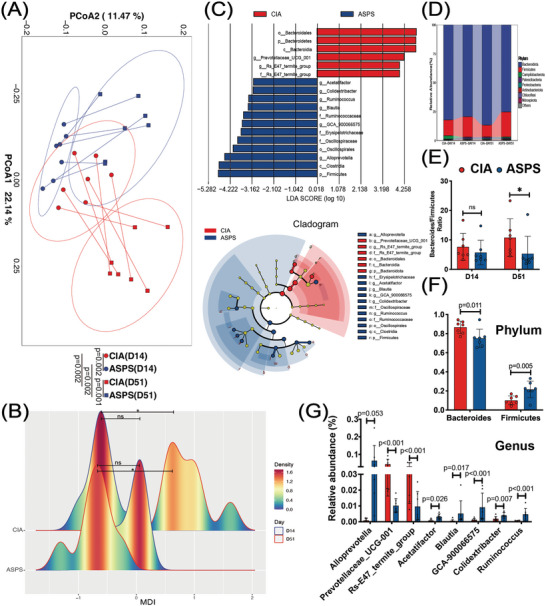
ASPS treatment reprogrammed the gut microbiota of CIA mice. A) Bray–Curtis distance‐based principle coordinate analysis (PCoA) plot from mice in CIA and ASPS groups on the 14th and 51st day after primary immunization. (*n* = 7). B) The microbial dysbiosis index (MDI) of mice (*n* = 7). C) LEfSe analysis of microbial samples on the 51st day after primary immunization with cutoff LDA = 3 (*n* = 7). D) Sankey diagram showing the relative abundance of TOP8 phyla (*n* = 7). E) The ratio of *Bacteroides*/*Firmicutes* (*n* = 7). F,G) Relative abundance of differential gut microbes identified by LEfSe analysis of microbial samples on the 51st day after primary immunization at phylum, and genus level (*n* = 7). Statistical analyses were performed with PERMANOVA (A), Kruskal–Wallis one way analysis of variance on ranks followed by the post hoc Tukey's comparisons (B,E), unpaired *t*‐test (F), or Mann–Whitney *U*‐test (G). Error bars indicate SEM.

Encouraged by the association between the anti‐rheumatic benefits of ASPS and gut microbiota, 16S rRNA sequencing of fecal microbial samples was used to determine further the gut microbiota changes triggered by arthritic progression and ASPS treatment. We found that ASPS treatment slightly raised the alpha diversity of gut microbiota in mice, while the arthritic progression further reduced the alpha diversity at the end of the treatment period in both ASPS‐treated mice and CIA mice (Figure S6A, Supporting Information). We use betadisper, a multivariate analogue of Levene's test for homogeneity of variances, to determine the homogeneity of dispersion among groups. The results showed that dispersion among groups was not significant (Figure S6B, Supporting Information, *F* = 1.85, *p* = 0.171, tested by PERMANOVA), which meets the "one assumption" for the adonis test. Weiter, Principal Coordinate Analysis (PCoA) based on Bray–Curtis distance reveals the differential clustering of gut microbial communities among groups (**Figure** **3**A, *R* ^2^ = 0.356, *p* = 0.001, tested by PERMANOVA). The beta diversity between CIA and ASPS‐treated mice was significantly different whether on the 14th or 51st day after the primary immunization (*R* ^2^ = 0.164, *p* = 0.002 for D14; *R* ^2^ = 0.346, *p* = 0.001 for D51). Interestingly, both arthritic progression and the continuous ASPS administration had contributed to a further increase in the difference in beta diversity (*R* ^2^ = 0.281, *p* = 0.002 for CIA [D14] vs CIA [D51]; *R* ^2^ = 0.206, *p* = 0.002 for ASPS [D14] vs ASPS [D51]), leading to the amplified difference between CIA and ASPS‐treated mice. Similarly, the microbial dysbiosis index (MDI) showed no significant difference between CIA and ASPS‐treated mice on the 14th day after primary immunization. With the arthritic progression, the MDI of CIA mice increased (CIA [D14 vs CIA [D51] *p* < 0.05), whereas ASPS treatment decreased the MDI (ASPS [D14 vs ASPS [D51] *p* > 0.05), making a significant difference between CIA and ASPS‐treated mice on the 51st day after primary immunization (*p* < 0.05, Figure 3B). Linear discriminant analysis Effect Size (LEfSe) analysis (LDA cutoff = 3) identified 12 strains of bacteria enriched in ASPS‐treated mice on the 51st day after primary immunization, while six strains were enriched in CIA mice (Figure 3C and Figure S6C, Supporting Information). Further analysis showed that although *Bacteroides* was the most dominant phylum in all groups, ASPS treatment significantly reversed the increased trend in abundance of *Bacteroides* and the ratio of *Bacteroides*/*Firmicutes* caused by arthritic progression (Figure 3D–F). Similar results were observed at class, order, and family levels (Figure S6D–F, Supporting Information). Especially, we found that *Ruminococcus*, *GCA‐900066575*, *Colidextribacter*, *Blautia*, and *Acetatifactor* were significantly enriched in ASPS‐treated mice at the genus level (Figure 3G).

The gut microbiota changes triggered by arthritic progression and fecal microbiota transplantation were determined by 16S rRNA sequencing as well. The results of betadisper showed that dispersion between groups meets the "one assumption" for the adonis test (Figure S7A, Supporting Information, *F* = 0.0105, *p* = 0.877, tested by PERMANOVA). PCoA analysis based on Bray–Curtis distance reveals the differential clustering of gut microbial communities between groups (Figure S7B, Supporting Information, *R* ^2^ = 0.188, *p* = 0.009, tested by PERMANOVA). LEfSe analysis (LDA cutoff = 3) identified four strains of bacteria enriched in ASPS‐trans mice on the 51st day after primary immunization, while three strains were enriched in CIA‐trans mice (Figure S7C, Supporting Information). *Bacteroides* was the most dominant phylum in both groups. ASPS‐trans treatment significantly reversed the increased trend in abundance of *Bacteroides* and the ratio of *Bacteroides*/*Firmicutes* caused by arthritic progression (Figure S7D, Supporting Information). We discovered that the ASPS‐trans mice also had a genus‐level enrichment of *Ruminococcus, GCA‐900066575, Colidextribacter, Blautia*, and *Acetatifactor*. (Figure S7E, Supporting Information). All these results were consistent with the changes in gut microbiota in CIA mice after ASPS treatment.

### 2.4 ASPS Raised Serum *γ*‐Glutamylcysteine Concentration

**Figure 4:**
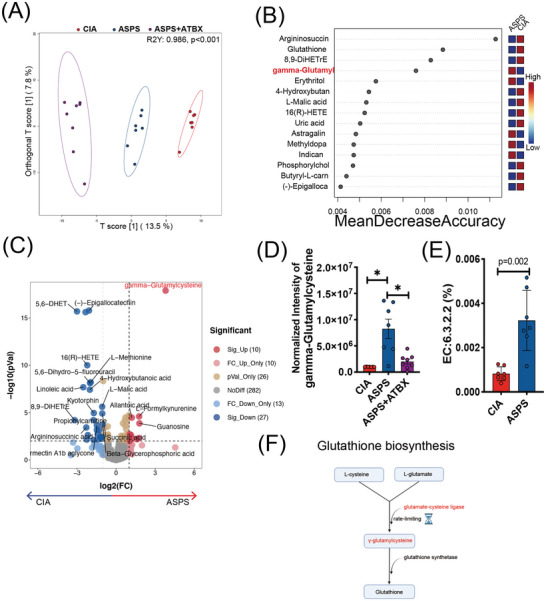
ASPS treatment enriched the level of serum *γ*‐glutamylcysteine. A) The OPLS‐DA plot of the serum metabolome (MS2 features). (*n* = 7 or 8). B) Random forest plot of MS2 features, ranking by the classification accuracy contribution. C) Volcano plot showing significant DMPs in the serum of CIA mice with/without ASPS treatment. D) Normalized intensity of serum *γ*‐glutamylcysteine (*n* = 7 or 8). E) Relative abundance of the *γ*‐glutamylcysteine synthesis‐related enzyme (EC: 6.3.2.2) (*n* = 7). F) *γ*‐glutamylcysteine in Glutathione biosynthesis.Figure created with BioRender.com. Statistical analyses were performed with Kruskal–Wallis one way analysis of variance on ranks followed by the post hoc Dunn's comparisons (E), or Mann–Whitney *U*‐test (F). Error bars indicate SEM.*p < 0.05.

To directly evaluate the influence of gut microbiota on the arthritic progression of CIA mice, we performed untargeted UPLC‐MS metabolomics using serum samples of CIA mice. The PCA plots showed that the QC samples in both ESI+ and ESI− models were closely clustered, indicating good repeatability and a stable system (Figure S9A,B, Supporting Information). The orthogonal partial least‐squares discrimination analysis (OPLS‐DA) showed apparent separation between CIA, ASPS, and ASPS + ATBX groups in both ESI^+^/^−^ and MS1/2 features (**Figure** **4**A and Figure S9C–E, Supporting Information). There were 269/17004, 182/9228, 372/2211, and 62/371 significant differential ESI^+^, ESI^−^, MS1, and MS2 features according to selection criteria of adjusted One‐way ANOVA [post hoc: Fisher's LSD] *p*‐value < 0.05, and the variable importance in the projection (VIP) > 1. The TOP5 enriched metabolic pathways included alanine, aspartate, and glutamate metabolism, arachidonic acid metabolism, citrate cycle, glutathione metabolism, and pyruvate metabolism (Figure S9F,G, Supporting Information). Of interest, 14 metabolic features were significantly enriched in glutathione metabolism. Interestingly, the important metabolic intermediate (GGC), which was synthesized by glutamate cysteine ligase (the rate‐limiting enzyme of glutathione synthesis), was enriched in ASPS‐treated mice compared with CIA mice (Figure 4B–D,F and Figure S7, Supporting Information). Furthermore, its intensity was decreased in antibiotic cocktail‐treated mice. Encountered with this interesting phenomenon, PICRUSt2^[^ ^29^ ^]^ was used to analyze the metabolic function profile. Amazingly, glutamate cysteine ligase (EC:6.3.2.2) was significantly enriched by ASPS treatment (Figure 4E). Taken together, GGC may act as a metabolite of gut microbiota and may be responsible for the anti‐rheumatic benefits of ASPS.

### 2.5 Supplement of *γ*‐Glutamylcysteine Could Alleviate RA Symptoms in CIA Mice Model

**Figure 5:**
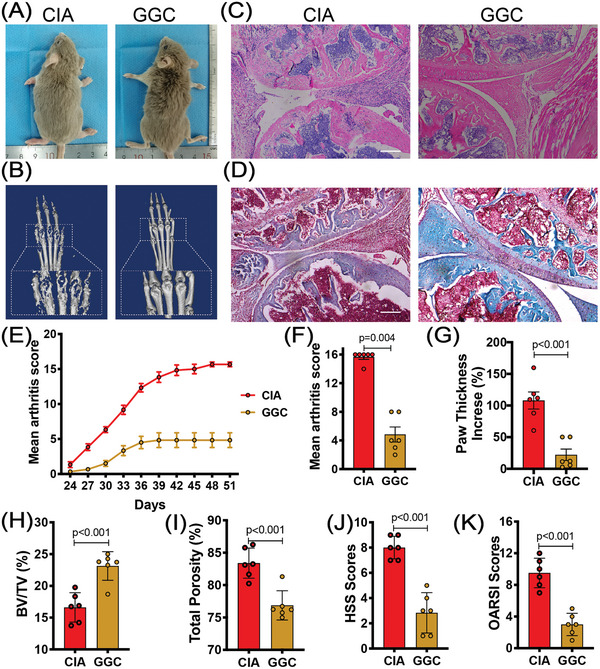
*γ*‐glutamylcysteine (GGC) could effectively suppress inflammatory arthritis in CIA mice. 8‐week‐old male DBA/1 mice were divided into two groups: CIA (collagen‐induced arthritis model), and GGC (CIA mice treated with GGC at a dose of 100 mg kg^−1^ per day from the primary immunization). A) Representative view of mice on the 51st day after primary immunization (sacrifice). B) Representative micro‐CT reconstruction image of the hind paws. C,D) Representative H&E stained, and Safranin O stained images of knee joints. Scale bar: 100 µm. E) Clinical arthritis score of the mice (*n* = 6). F) Clinical arthritis score of the mice on the 51st day after primary immunization (*n* = 6). G) Hind paw thickness changes (*n* = 6). H,I) Quantitative analysis of BV/TV, and total porosity (*n* = 6). J) Histological synovitis scores (HSS) of knee joints (*n* = 6). K) Modified OARSI scores of knee joints (*n* = 6). Statistical analyses were performed with *t*‐test (G–K). Error bars indicate SEM.

As mentioned above, ASPS treatment can increase the concentration of GGC in the serum of CIA mice. But, whether GGC mediated the anti‐rheumatic effects of ASPS was still elusive. Then, the influence of GGC was further investigated in the CIA model. As expected, GGC‐treated mice exhibited attenuated arthritis symptoms, similar to ASPS treatment. The arthritis score and paw thickness increase were suppressed (**Figure** **5**A,E,F,G). The bone erosion activity was effectively inhibited by GGC (Figure 5B,H,I). We also observed relived cartilage damage and pannus formation in GGC‐treated mice (Figure 5C,D,J,K). Thus, we could conclude that the protective effect of ASPS against RA may depend on the enrichment of GGC.

### 2.6 GGC Disrupts NLRP3 Inflammasome Activation via Inhibiting ASC Nucleation

**Figure 6:**
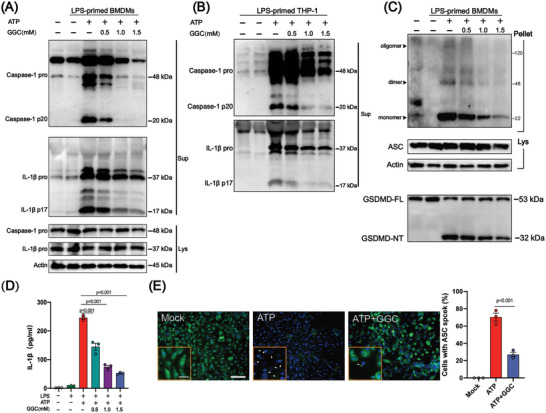
*γ*‐glutamylcysteine interrupted NLRP3 inflammasome activation by inhibiting ASC nucleation. Before stimulation with A,C–E) ATP, LPS‐induced BMDM or B) THP‐1 cells were treated with *γ*‐glutamylcysteine. A) Pro‐caspase‐1, cleaved‐caspase‐1 (p20), pro‐IL‐1*β*, and cleaved IL‐1*β* (p17) were detected in the supernatant (Sup) and cell extracts (Lys). B) Pro‐caspase‐1, pro‐IL‐1*β* were detected in cell lysates and cleaved‐caspase‐1 (p20), cleaved IL‐1*β* (p17) were detected in the supernatant (Sup). C) ASC oligomerization in cross‐linked cytosolic pellets was detected. D) IL‐1*β* release in supernatants was detected by ELISA. E) Representative immunofluorescence images and quantification of ASC‐speck formation. Scale bar: 100 (outer) or 10 µm (inner). Statistical analyses were performed with ANOVA followed by the post hoc Student–Newman–Keuls pairwise comparisons (D). Error bars indicate SEM.

**Figure 7:**
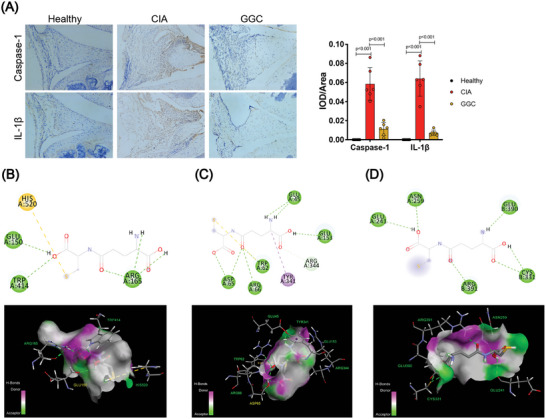
*γ*‐glutamylcysteine suppressed inflammatory arthritis via interrupting NLRP3 inflammasome activation. A) Representative immunohistochemical staining and quantification of Caspase‐1, IL‐1*β*. Scale bar: 100 µm. B–D) Optimal conformation of molecular docking between *γ*‐glutamylcysteine (GGC) with NLRP3, ASC, and Caspase‐1. Statistical analyses were performed with ANOVA followed by the post hoc Student–Newman–Keuls pairwise comparisons (A). Error bars indicate SEM.

NLRP3 inflammasome activation has been shown to be an important trigger for arthritis progression.^[^ ^30^ ^]^ The expression of NLRP3 inflammasome‐related genes was elevated in RA patients. The arthritic symptoms could be effectively suppressed either by inhibiting NLRP3 inflammasome activation or knockout NLRP3 inflammasome‐related genes. Hence, NLRP3 inflammasome is a potential rational therapeutic target for RA treatment. Encouraged by the result that GGC could alleviate RA symptoms, we intended to figure out whether GGC exerted the anti‐rheumatic effect via suppressing NLRP3 inflammasome activation. We treated lipopolysaccharide (LPS)‐primed bone‐marrow‐derived macrophages (BMDMs) with GGC before ATP stimulation. As expected, we found that GGC inhibited caspase‐1 activation and IL‐1*β* maturation in a dose‐dependent manner (**Figure** **6**A). Similar results were also observed in THP‐1 cells, that is, GGC inhibits NLRP3 inflammasome activation (Figure 6B). ASC oligomerization is one of the most common mechanisms of NLRP3 inflammasome activation. Our results indicated that GGC significantly attenuated the formation of ASC‐complex (Figure 6C). Moreover, ATP stimulated the NLRP3 inflammasome to cause ASC to condense into a large cytoplasmic speck, while GGC inhibited the formation of ASC‐speck in BMDMs (Figure 6E). NLRP3‐dependent caspase‐1 cleavage could specifically cleave GSDMD, which controls IL‐1*β* release.^[^ ^31^, ^32^, ^33^ ^]^ Correspondingly, GSDMD cleavage and IL‐1*β* secretion were also blocked by GGC in a dose‐dependent manner (Figure 6C,D). The expression levels of caspase‐1 and IL‐1*β* were also detected in joint specimens. The expression levels of caspase‐1 and IL‐1*β* remarkably decreased by more than 80% in GGC treated mice (**Figure** **7**A). Next, we further performed molecular docking study between GGC with the three targets NLRP3, ASC and Caspase‐1. The molecular docking result showed that the binding free energy of GGC with NLRP3 protein (PDB ID: 6NPY), ASC protein (PDB ID: 6KI0), Caspase‐1 protein (PDB ID: 2FQQ) were −5.7, −5.6, and −3.7 kcal mol^−1^, respectively, indicating that GGC can bind to NLRP3, ASC, Caspase‐1, and achieved favorable interactions. (Figure 7B–D and Table S1, Supporting Information). In addition, the level of IL‐1*β* in serum was further measured by ELISA method, which also proved that GGC could significantly reduce the level of inflammatory cytokines IL‐1*β* in serum (Figure S10D, Supporting Information). Compared with CIA mice, GGC treatment decreased the expression of F4/80, caspase‐1, and IL‐1*β* in macrophage‐expressing regions, indicating it could inhibit inflammasome activation in vivo (Figure S10A–C, Supporting Information). Hence, these results indicate that GGC interrupts NLRP3 inflammasome activation by inhibiting ASC nucleation.

## 3 Discussion

Polysaccharide is a class of natural polymeric polymers linked by glycosidic bonds and is widely found in aqueous extracts of Chinese herbal medicines. *A. senticosus* is a well‐known herbal medicine that contains many types of compositions, including polysaccharide, flavonoids, and triterpene saponins. *A. senticosus* extract can inhibit RANKL‐induced osteoclastogenesis by inhibiting the RANK signaling pathway, thereby improving bone loss induced by ovariectomy.^[^ ^34^ ^]^ In addition, several studies have shown the ameliorative effects of *A. senti*cosus on RA.^[^ ^13^, ^14^, ^15^ ^]^ ASPS is one of the most important active components of *A. senticosus*. However, the application of ASPS in the treatment of RA has never been reported, and whether ASPS exhibit osteoprotective and anti‐rheumatic effects were also urgently needed further investigation. Therefore, we carried out the study to explore the positive impacts of ASPS on RA and the possible molecular mechanisms behind them. In this study, we proved that oral ASPS was effective in suppressing rheumatic inflammation in CIA mice with low levels of arthritis symptoms, bone erosion activity, cartilage damage, and pannus formation.

Systemic homeostasis is closely related to the gut microbiota, especially in the immune system.^[^ ^35^ ^]^ Gut microbiota is considered to be one of the most relevant environmental factors influencing the development of CIA. Recently, the polysaccharide has been reported to have the ability to regulate gut microbial ecology, and ASPS has been shown to attenuate intestinal tight junction damage by inhibiting the NF‐*κ*B/MLCK pathway.^[^ ^20^ ^]^ Oral ASPS administration improves growth performance, immune status, and antioxidant capacity of broilers and stimulates the growth of beneficial intestinal bacteria.^[^ ^36^ ^]^ However, it is unclear whether ASPS can exert anti‐rheumatic effects through the gut microbiota. Our experimental results showed severer arthritis in CIA mice when treated with antibiotic cocktail therapy, implying that the removal of gut microbiota exacerbates rheumatoid arthritis. Furthermore, it was showed that the protective effect of ASPS on joints was diminished when CIA mice were given ASPS and antibiotic cocktails simultaneously. These results indicated that the anti‐rheumatic effects of ASPS are mediated through the gut microbiota. We then performed a fecal microbiota transplantation experiment to further validate our results and found that it could mimic the anti‐rheumatic effect similar to ASPS treatment. Encouraged by these results, we hypothesized that the anti‐rheumatic benefits of ASPS were mediated by gut microbiota

Interestingly, marked changes in gut microbiota were observed in ASPS‐treated mice. The results of 16S rRNA sequencing revealed that *Ruminococcus, GCA‐900066575, Colidextribacter, Blautia*, and *Acetatifactor* were significantly enriched in ASPS‐treated mice. Gut microbes could produce a variety of metabolites that are essential for regulating multiple host‐microbiome pathways, such as short‐chain fatty acids (SCFA), LPS, and propionate imidazole.^[^ ^27^, ^37^, ^38^, ^39^ ^]^ Hence, we performed untargeted UPLC‐MS metabolomics using serum samples to identify differential metabolites that may be responsible for the anti‐rheumatic benefits of ASPS. 14 metabolic features were significantly enriched in glutathione metabolism, which was one of the TOP5 enriched metabolic pathways. Of note, GGC, the important metabolic intermediate of the glutathione metabolism pathway, was significantly enriched by ASPS treatment and depleted by antibiotic cocktail treatment. Moreover, considering the correlation between gut microbiota and metabolites, we found that glutamate cysteine ligase was also enriched by ASPS treatment using PICRUSt2, an accurate approach of functional genes prediction for 16S rRNA. These results indicated that GGC may act as a metabolite of gut microbiota and be responsible for the anti‐rheumatic benefits of ASPS. GGC is an important intermediate metabolite in glutathione synthesis pathway and exhibits antioxidant effects due to its cysteine residues. It can increase brain glutathione levels and improve oxidative damage in neurons and astrocytes.^[^ ^40^ ^]^ Bifidobacterium dentium‐derived GGC has been reported to inhibit endoplasmic reticulum‐mediated cup cell stress and reduce TNBS‐driven colonic inflammation, resulting in positive regulation of the unfolded protein response (UPR) and MUC2 production.^[^ ^41^ ^]^ In vitro studies have also shown that the addition of exogenous GGC can resolve cellular markers of oxidative stress.^[^ ^42^, ^43^ ^]^ Yang et al. discovered the anti‐inflammatory benefits of GGC, with the evidence that GGC protected mice from CLP‐ or LPS‐induced lethal toxicity.^[^ ^44^ ^]^ The impressive anti‐inflammatory effect of GGC implies the potential therapeutic benefits of GGC in RA treatment. Therefore, we further treated the CIA mice with oral GGC administration. Consistent with our hypothesis, GGC exhibited a good therapeutic effect against CIA. So far as we know, this is the first time to identify the therapeutic potential of GGC in CIA.

NLRP3 inflammasome activation has been shown to be an important trigger for arthritis progression.^[^ ^30^ ^]^ Compared with healthy controls, the gene expression of NLRP3, ASC, caspase‐1, IL‐1*β*, and IL‐1R in the PBMC of RA patients was significantly higher.^[^ ^45^, ^46^, ^47^ ^]^ Single nucleotide polymorphisms in the NLRP3 locus are associated with higher RA susceptibility and disease activity.^[^ ^45^, ^47^ ^]^ Especially, both serum and synovial fluid IL‐1*β* increase in RA patients with active stage.^[^ ^48^ ^]^ The clinical score of arthritis is positively correlated with the level of NLRP3 in synovial tissue.^[^ ^49^ ^]^ By inhibiting the NLRP3 inflammasome in macrophages, the arthritic symptoms can be effectively suppressed.^[^ ^50^ ^]^ ASC knockout could protect mice from developing arthritis in CIA model.^[^ ^51^ ^]^ Knockout of caspase‐1, IL‐1*β*, or IL‐1 receptor could also diminish RA‐associated inflammation and joint destruction.^[^ ^30^, ^51^, ^52^, ^53^ ^]^ Our results also confirmed that NLRP3 inflammasome activation was highly promoted in CIA mice. Although several studies have confirmed the anti‐inflammatory effect of GGC.^[^ ^44^ ^]^ As far as we know, there is still no report that GGC could inhibit the expression of NLRP3 inflammasome. In this study, we identified that GGC could evidently block caspase‐1 maturation and IL‐1*β* secretion in BMDMs and THP‐1 macrophages. Meanwhile, the decreased level of caspase‐1 and IL‐1*β* expression could be observed in GGC‐treated mice's joint specimens via immunohistochemistry staining and immunofluorescence staining. More importantly, we identified that GGC disrupted ASC oligomerization and speck formation. The mechanism that GGC interrupted NLRP3 inflammasome activation via inhibition of ASC nucleation was initially verified in this study. As discussed above, we can conclude that the therapeutic effect against RA of ASPS is potentially mediated by modulating the gut microbiota and its metabolite GGC.

## 4 Conclusions 

Taken together, the anti‐rheumatic effect of ASPS is initially verified in CIA model. In addition, it is the first to fully reveal the mechanism by which ASPS reverses the dysbiosis triggered by arthritic progression, enhances GGC synthetase expression, and increases serum GGC concentration. Moreover, GGC mediated inhibition of NLRP3 inflammasome activation may be responsible for the anti‐rheumatic benefits of ASPS. Thus, our study highlighted the therapeutic potential of ASPS against CIA, which mainly exhibited its anti‐rheumatic effects via modulating gut microbiota and regulating GGC production.

## 5 Experimental Section

Provided in the Supporting Information.

## Conflict of Interest

The authors declare no conflict of interest.

## Author Contributions

A.L., M.Z., Y.W. contributed equally to this work. J.L., C.H., and Z.Y. designed the research; A.L., M.Z., and Y.W. performed the experiments; W.S., X.Z., X.S., J.L., and Y.Z. analyzed the data; Z.Y. and J.L. wrote the paper. All authors approved the final version to be published.

## Supporting information

Supporting Information

## Data Availability

The data that support the findings of this study are available from the corresponding author upon reasonable request.
