# Supplementary material for: ASPS Exhibits Anti‐Rheumatic Effects by Reprogramming Gut Microbiota and Increasing Serum γ‐Glutamylcysteine Level
Source: Adv Sci (Weinh). 2022 Nov 23;10(3):2205645. doi: 10.1002/advs.202205645 (PMC9875676; doi:10.1002/advs.202205645)
Supplement: Supplementary file 1 — Supporting Information [file ADVS-10-2205645-s001.pdf]

ADVANCED SCIENCE
Open Access

Supporting Information

for *Adv. Sci.*, DOI 10.1002/advs.202205645

ASPS Exhibits Anti-Rheumatic Effects by Reprogramming Gut Microbiota and Increasing Serum γ-Glutamylcysteine Level

Ang Liu, Min Zhang, Yanglin Wu, Chenhui Zhang, Qin Zhang, Xinlin Su, Xu Zhu, Weidong Shi, Jiangyun Liu, Yang Zhang, Cheng Huang, Zhaowei Yan* and Jun Lin**

ASPS exhibits anti-rheumatic effects by reprogramming gut microbiota and increasing serum γ-glutamylcysteine level

Running title: ASPS alleviates CIA via γ-glutamylcysteine

Ang Liu[1,2†], Min Zhang[3, 4†], Yanglin Wu[1,5†], Chenhui Zhang[1,2], Qin Zhang[1,2], Xinlin Su[2], Xu Zhu[2], Weidong Shi[2], Jiangyun Liu[4], Yang Zhang[6], Cheng Huang[7*], Zhaowei Yan[3, 4*], Jun Lin[1*]

[1] Department of Orthopaedics, Suzhou Dushu Lake Hospital, Dushu Lake Hospital Affiliated to Soochow University, Medical Center of Soochow University, Suzhou, 215125, China

[2] Department of Orthopaedics, The First Affiliated Hospital of Soochow University, Suzhou, 215006, China

[3] Department of Pharmacy, The First Affiliated Hospital of Soochow University, Suzhou, 215006, China

[4] College of Pharmaceutical Sciences, Soochow University, Suzhou, 215123, China

[5] Department of Orthopaedics, Shanghai Tenth People's Hospital, School of Medicine, Tongji University, Shanghai, 200092, China.

[6] School of Biology and Food Engineering, Changshu Institute of Technology, Changshu, 215500, China

[7] Department of Orthopaedics, China-Japan Friendship Hospital, Beijing, 100029, China

[†] These authors contributed equally.

Corresponding Authors:

[*] Jun Lin, M.D., Ph.D., Department of Orthopaedics, Suzhou Dushu Lake Hospital,

Dushu Lake Hospital Affiliated to Soochow University, Medical Center of Soochow University, Suzhou, 215125, China, Email: linjun@suda.edu.cn

* Zhaowei Yan, Ph.D., Department of Pharmacy, The First Affiliated Hospital of Soochow University, Suzhou, 215006, China. Email: yanzwsuzhou@163.com

* Cheng Huang, M.D., Ph.D., Department of Orthopaedics, China-Japan Friendship Hospital, Beijing, 100029, China. Email: hcheng90@outlook.com

Methods

1. Purification and characterization of ASPS

The method to obtain polysaccharide is referred to Xia et al.[1] with some modifications. Briefly, the crude polysaccharide was obtained from *Acanthopanax senticosus* by hot-water extraction, ethanol precipitation, deproteinized by Sevag method, dialyzed with water, and lyophilized in freeze-dry apparatus. It was sequentially purified with a DEAE Cellulose-52 column (4 cm × 60 cm), and a concentration gradient of NaCl solutions (0-0.4 M) was used to elute the crude polysaccharide. The dominant fraction was collected for further purification on Toyopearl HW-55F gel-filtration column (3 cm × 60 cm). The homogeneity and molecular weight of ASPS were measured using high-performance gel permeation chromatography (HPGPC) on HPLC system (Shimadzu LC-20A, Japan) equipped with a refractive index detector.[2] ASPS was identified using an FT-IR spectrophotometer (VERTEX 70+HYPERION 2000, Germany) within 4000-400 cm^{-1}. In addition, the monosaccharide composition of ASPS was further determined by PMP derivatization.[3] And the morphology feature of ASPS was analyzed on a scanning electron microscope (SEM, Hitachi SU 8100, Japan).

2. Animal experiment

DBA/1 male mice aged 8 weeks were purchased from Beijing Charles River Company (Beijing, China) and kept under pathogen-free conditions with adequate food and water in the SPF animal laboratory. All animal experiments were conducted

with the permission of the ethical committees on laboratory animal welfare of Soochow University.

In the study of ASPS treatment, 32 mice (20.0 ± 0.3 g) were randomly divided into the healthy group, CIA group (collagen-induced arthritis model), ASPS group (CIA mice treated with ASPS per day from the primary immunization), and ASPS+ATBX group (CIA mice simultaneously treated with ASPS and antibiotic cocktail per day from the primary immunization). Mice in the ASPS and ASPS+ATBX groups were orally given 300 mg/kg of ASPS per day from the primary immunization, according to previous studies. [4,5] Mice in the ASPS+ATBX group were treated with ASPS and antibiotic cocktail. The antibiotic cocktail was diluted with 0.5 g/L ampicillin, 1 g/L streptomycin, 0.5 g/L Vancomycin, and 1 g/L gentamicin. All the antibiotics were purchased from Sigma-Aldrich.

In the study of fecal microbiome transplantation, mice were randomly divided into three groups: CIA group, ASPS-trans group (fecal microbiome transplantation of CIA mice treated with ASPS), and CIA-trans group (fecal microbiome transplantation of CIA mice). Initially, each group received the same mixture of antibiotics for drinking water. The concentration of antibiotics cocktails was 0.5 g/L ampicillin, 1 g/L streptomycin, 0.5 g/L Vancomycin, and 1 g/L gentamicin and the water were changed every week. After two weeks, the antibiotic water was replaced with normal water. CIA-trans and ASPS-trans groups were then given 0.2 ml fecal solution from CIA and ASPS groups by gavage once a day. For the FMT experiments, briefly, fecal samples were collected from CIA and ASPS groups. About 100 mg feces was re-suspended in

3.0 ml sterile PBS. Fecal samples were well mixed and then centrifuged at 6000 rpm for 10 min. The microbiota supernatants from CIA and ASPS groups were respectively transplanted into the CIA-trans and ASPS-trans groups. Simultaneously with the start of FMT treatment, mice were induced to CIA models. After completing 51 consecutive days of microbiome transplantation, they were killed and then subjected to radiological and histological analysis.

In the GGC treatment study, mice were randomly assigned to the CIA group and the GGC (CIA mice treated with GGC at a dose of 100 mg/kg per day from the primary immunization) group. The GGC (Shanghai yuanye Bio-Technology Co., Ltd) oral consumption in this study was given at a dose of 100 mg/kg of body weight per day according to the previous study which used an allometric scaling calculator (http://clymer.altervista.org/minor/allometry.html) to calculate the appropriate dosage using information from the clinic. [6] The control group was orally administered the same volume of blank solvent water without GGC.

Stool, serum, joint, and paw samples from each group were collected for further analysis during the study.

3. Collagen-induced arthritis model

The CIA mouse model was established by a double immunization. For the first immunization, mice were injected subcutaneously at the end of the tail with an emulsion of equal volume bovine type-II collagen solution (2 mg/mL) and completed Freund's adjuvant (4 mg/mL). After 21 days of the first immunization, the boost

immunization was given to the mice with bovine type-II collagen solution emulsified in incomplete Freund's adjuvant.

4. Clinical scoring and paw thickness measurement

The mice were scored once three days after the second immunization. A score of 0–4 was assigned to each hind paw according to the previous study[7] as follows: 0, normal; 1, swelling of toes; 2, swelling of the ankle and/or tarsus; 3, moderate swelling of the ankle and/or tarsus or mild swelling of both and 4, severe swelling of the entire paw. The thickness of the paw was also measured before primary immunization (DAY0) and on the 51st day after primary immunization (DAY51) using a caliper. Paw thickness increase was defined as (Thickness^{DAY51} – Thickness^{DAY0})/Thickness ^{DAY0}×100 (%).

5. Cell culture and stimulation

Bone marrow-derived macrophages were isolated from both femurs and tibias of mice. Bone marrow was flushed from the cut ends of the femur and tibia. The bone marrow fluid was taken for the hemolysis test. Bone marrow cells were then re-suspended in DMEM with 10% FBS and 50 ng/ml M-CSF (R&D Systems, USA) and placed in a twelve-well plate with 2–4 × 10^6 cells/ml. The cells were then cultured for five to seven days for further study. To activate the inflammasome in BMDMs, the cells were incubated with GGC and 500 ng/ml LPS for 4 h and then stimulated with 3mM ATP for 1h. GGC was administered at a concentration of 0.5mM, 1.0mM, and 1.5mM.

6. Western blot assay

Cells were lysed in RIPA lysis buffer (Beyotime, China) containing protease and phosphatase inhibitor, and protein concentration was measured by a BCA protein assay kit (Novoprotein, Shanghai, China). The supernatant proteins were precipitated. The protein was separated by SDS-PAGE (Beyotime, China) and transferred to the polyvinylidene fluoride membrane (Beyotime, China). The cell membranes were cultured overnight with anti-procaspase1, anti-cleaved caspase-1, anti-caspase-1 pro, anti-ASC, anti-IL 1β (Cell Signaling Technology, MA, USA), anti-GSDMD (Abcam, USA) and anti-Actin (Beyotime, China) at 4 ° C following the block with quick block buffer (Beyotime, China) for thirty minutes. After three rinses with TBS-Tween, the membrane was placed in the secondary antibody for one hour at room temperature. The results were visualized via chemiluminescent peroxidase substrate (Sigma-Aldrich). The specific information of primary antibodies used in WB is as follows: Caspase-1 (1:1000, AG-20B-0042-C100, Adipogen), IL-1β (1:1000, 27989, Cell signal technology) used in supernatants. Caspase-1 pro (1:500, sc-56036, Santa Cruz Biotechnology), IL-1β pro (1:500, sc-52012, Santa Cruz Biotechnology), ASC (1:1000, 67824, Cell signal technology), Actin (1:1000, AA128, Beyotime) used in cell lysates.

7. Micro-CT analysis

Hind paws collected from every group were subjected to Micro-CT (micro-computed tomography) scanning (SkyScan 1176, Aartselaar, Belgium). The parameters of the X-Ray were set at a current of 500 μA with a voltage of 50 kV and the scanning per layer was 9 μm. The software CTAn was applied to analyse the hind

paws for bone volume to tissue volume ratio (BV/TV, %), trabecula number (TB.N, mm), trabecula thickness (TB.Th, 1/mm), total porosity (%), Bone surface (BS, mm^2), and Moment of inertia (MMI, mm^5).

8. Histology staining and immunohistochemistry staining

For the evaluation of the severity of inflammation, the mouse knee joints were embedded with paraffin. The specimen was cut to slices every 6 μm. H&E and safranin-O staining were guided by the manufacturer's instructions. For immunohistochemistry staining, knee joint sections were incubated with Caspase-1 (1:200, sc-56036, Santa Cruz Biotechnology) and IL-1β (1:200, sc-52012, Santa Cruz Biotechnology) at 4°C overnight. Then washed with PBS for three times, the slices were stained with second antibody for 1 h at room temperature and in 100μl DAB for 2-6 minutes.

9. Immunofluorescence staining

BMDMs were plated at a density of 1×10^6 /well in a twelve-well plate after LPS-priming and treated with inflammasome inducers in the presence of wedelolactone for the indicated time. To observe the formation of ASC speck, indicated reagents were used to treat the BMDMs. Macrophages were immobilized in 4% paraformaldehyde for 15 min and permeated with frozen methanol for 10 min. Then, cells were incubated with various primary antibodies at 4°C overnight, followed by staining with Alexa Fluor 488 goat anti-rabbit IgG or Alexa Fluor 555 goat anti-mouse IgG. The nucleus was stained with DAPI (KeyGEN, China). Fluorescence images were observed under Zeiss LSM 800.

Knee joint sections were incubated with Caspase-1(mouse, Proteintech), IL-1β (mouse, Proteintech), and F4/80 (rabbit, Abcam) at 4°C overnight. Following washing with PBS three times, slices were incubated with Goat Anti-Mouse IgG H&L (Alexa Fluor® 488, Abcam), Goat Anti-Rabbit IgG H&L (Alexa Fluor® 647, Abcam), and DAPI (Keygen BioTECH, China) for 60 min and 10 min respectively.

10. Molecular docking

We used the molecular docking method to investigate the interaction potential and binding efficacy of GGC towards the three-dimensional structure of the most important protein molecules of NLRP3 inflammasomes, including NLRP3, ASC, and Caspase-1. ChemBio3D Ultra 14.0 program was used to construct 3D structures of GGC, energy minimized, and converted to mol2 files. Afterward, the X-ray crystal structures of NLRP3 protein (PDB ID: 6NPY), ASC protein (PDB ID: 6KI0) and Caspase-1 protein (PDB ID: 2FQQ) were obtained from RCSB Protein Data Bank (www.rcsb.org/pdb). These structures were loaded into the AutoDock Tools 1.5.6 program [8], the atomic charge was added, the atomic type was assigned, and all flexible bonds could be rotated by default. The binding mechanism of NLRP3, ASC, and Caspase-1 to GGC was analyzed using Auto Dock Vina 1.1.2.[9] The binding energies and contacts of the ligand were obtained. Finally, the Discovery Studio 4.0 Visualizer (BIOVIA, CA, USA) was used for the visualization analysis of the results.

11. 16S rRNA gene sequencing

The sequencing library was prepared using TruSeq DNA PCR-Free sample preparation kit (Illumina, USA). An Illumina NovaSeq platform was used to sequence

the library (Wekemo Tech Co., Ltd. Shenzhen China).

12. Microbial dysbiosis index

The MDI (Microbial dysbiosis index) was used to evaluate microbial dysbiosis. The MDI was defined as the log value of [total abundance of bacterial genera enriched in CIA mice] divided by [total abundance of bacterial genera depleted in CIA mice].[10,11]

13. Untargeted metabolomics analysis

The serums of mice were transferred on dry ice to the BioNovoGene Metabonomics Platform, Suzhou.

All of them were defrosted at 4 °C and blended consistently (The insufficient samples are reduced to an equal scale). 35-50 μL of each sample was taken in a 2 mL microcentrifuge tube, to which 400 μL of methanol was added and vortexed for 60 s and centrifuged at 12,000 RPM at 4 °C for 10 min. The supernatants were exchanged to another 2 mL micro-centrifuge tube, dried and dissolve samples with 150 μL 2-chlorobenzalanine (4 ppm) 80% methanol solution.[12] Next, the supernatants were separated through a 0.22 μm film to acquire the examples for analysis. The samples were analyzed through ACQUITY UPLC® HSS T3 (150×2.1 mm, 1.8 μm, Waters) chromatography column and mass spectrometry using Thermo QE-HF-X instrument with electrospray ionization (ESI) and cation–anion ionization mode.[13,14]

14. Enzyme-linked immunosorbent assay

TNF-α in mice's serum, IL-1β in cell supernatants and serum were measured by ELISA kit (MultiSciences) following the manufacturer's instructions.

15. Statistical analysis

Sigmaplot V12.5, Prism V8.2.1, and R program V4.1.1 were applied to perform statistical analysis.

References

[1] Y. G. Xia, Y. X. Huang, J. Liang, H. X. Kuang, *Int. J. Biol. Macromol.* **2020**, *147*, 350.

[2] J. Zhu, W. Liu, J. Yu, S. Zou, J. Wang, W. Yao, X. Gao, *Carbohydr. Polym.* **2013**, *98*, 8.

[3] H. Zhao, J. Xu, R. Wang, W. Tang, L. Kong, W. Wang, L. Wang, Y. Zhang, W. Ma, *Food Funct.* **2021**, *12*, 2543.

[4] Q. Yang, Q. Wang, W. Deng, C. Sun, Q. Wei, M. Adu-Frimpong, J. Shi, J. Yu, X. Xu, *Int. J. Biol. Macromol.* **2019**, *123*, 801.

[5] Z. Z. Shang, D. Y. Qin, Q. M. Li, X. Q. Zha, L. H. Pan, D. Y. Peng, J. P. Luo, *Carbohydr. Polym.* **2021**, *258*, 117657.

[6] Y. Liu, Z. Chen, B. Li, H. Yao, M. Zarka, J. Welch, P. Sachdev, W. Bridge, N. Braidy, *Neurochem. Int.* **2021**, *144*, 104931.

[7] D. D. Brand, K. A. Latham, E. F. Rosloniec, *Nat. Protoc.* **2007**, *2*, 1269.

[8] G. M. Morris, R. Huey, W. Lindstrom, M. F. Sanner, R. K. Belew, D. S. Goodsell, A. J. Olson, *J. Comput. Chem.* **2009**, *30*, 2785.

[9] O. Trott, A. J. Olson, *J. Comput. Chem.* **2010**, *31*, 455.

[10] J. Lloyd-Price, C. Arze, A. N. Ananthakrishnan, M. Schirmer, J. Avila-Pacheco, T. W. Poon, E. Andrews, N. J. Ajami, K. S. Bonham, C. J. Brislawn, D. Casero, H. Courtney, A. Gonzalez, T. G. Graeber, A. B. Hall, K. Lake, C. J. Landers, H. Mallick, D. R. Plichta, M. Prasad, G. Rahnavard, J. Sauk, D. Shungin, Y. Vazquez-Baeza, R. A. White, 3rd, I. Investigators, J. Braun, L. A. Denson, J. K. Jansson, R. Knight, S. Kugathasan, D. P. B. McGovern, J. F. Petrosino, T. S. Stappenbeck, H. S. Winter, C. B. Clish, E. A. Franzosa, H. Vlamakis, R. J. Xavier,

C. Huttenhower, *Nature* **2019**, *569*, 655.

[11] D. Gevers, S. Kugathasan, L. A. Denson, Y. Vazquez-Baeza, W. Van Treuren, B. Ren, E. Schwager, D. Knights, S. J. Song, M. Yassour, X. C. Morgan, A. D. Kostic, C. Luo, A. Gonzalez, D. McDonald, Y. Haberman, T. Walters, S. Baker, J. Rosh, M. Stephens, M. Heyman, J. Markowitz, R. Baldassano, A. Griffiths, F. Sylvester, D. Mack, S. Kim, W. Crandall, J. Hyams, C. Huttenhower, R. Knight, R. J. Xavier, *Cell Host Microbe* **2014**, *15*, 382.

[12] A. Demurtas, S. Pescina, S. Nicoli, P. Santi, D. Ribeiro de Araujo, C. Padula, *J. Chromatogr. B. Analyt. Technol. Biomed. Life Sci.* **2021**, *1164*, 122512.

[13] O. H. Abdelhafez, E. M. Othman, J. R. Fahim, S. Y. Desoukey, S. M. Pimentel-Elardo, J. R. Nodwell, T. Schirmeister, A. Tawfike, U. R. Abdelmohsen, *Phytochem. Anal.* **2020**, *31*, 204.

[14] G. Monnerat, F. A. C. Seara, J. A. M. Evaristo, G. Carneiro, G. P. C. Evaristo, G. Domont, J. H. M. Nascimento, J. G. Mill, F. C. S. Nogueira, A. C. Campos de Carvalho, *J. Steroid Biochem. Mol. Biol.* **2018**, *183*, 39.

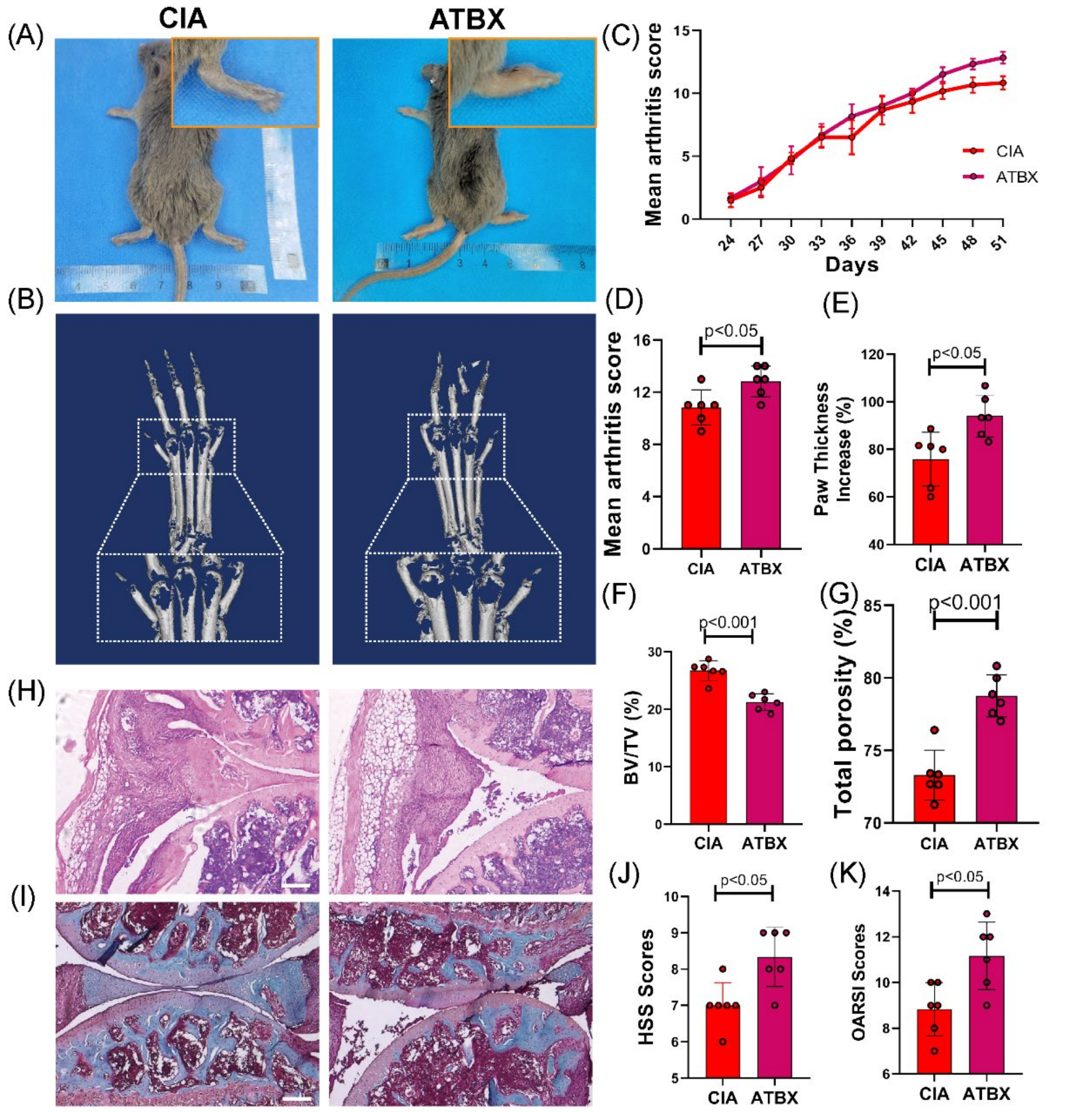

Figure S1. The removal of gut microbiota enhanced inflammatory arthritis in CIA mice. 8-week old male DBA/1 mice were divided into two groups: CIA (collagen-induced arthritis model), and ATBX (CIA mice only treated with antibiotic cocktail).

(A) Representative view of mice on the 51st day after primary immunization (sacrifice).

(B) Representative micro-CT reconstruction images of the hind paws with detailed interphalangeal joints images on the 51st day after primary immunization (sacrifice).

(C) Clinical arthritis score of the mice. (n = 6)

(D) Clinical arthritis score of the mice on the 51st day after primary immunization. (n = 6)

(E) Hind paw thickness changes. (n = 6)

(F) Quantitative analysis of BV/TV. (n = 6)

(G) Quantitative analysis of total porosity. (n = 6)

(H) Representative H&E stained images of knee joints. Scale bar: 50 μm.

(I) Representative Safranin O stained images of knee joints. Scale bar: 50 μm.

(J) Histological synovitis scores (HSS) of knee joints. (n = 6)

(K) Modified OARSI scores of knee joints. (n = 6)

Statistical analyses were performed with ANOVA followed by the post hoc Student-Newman-Keuls pairwise comparisons (D, E, F, G, J, K). Error bars indicate SEM.

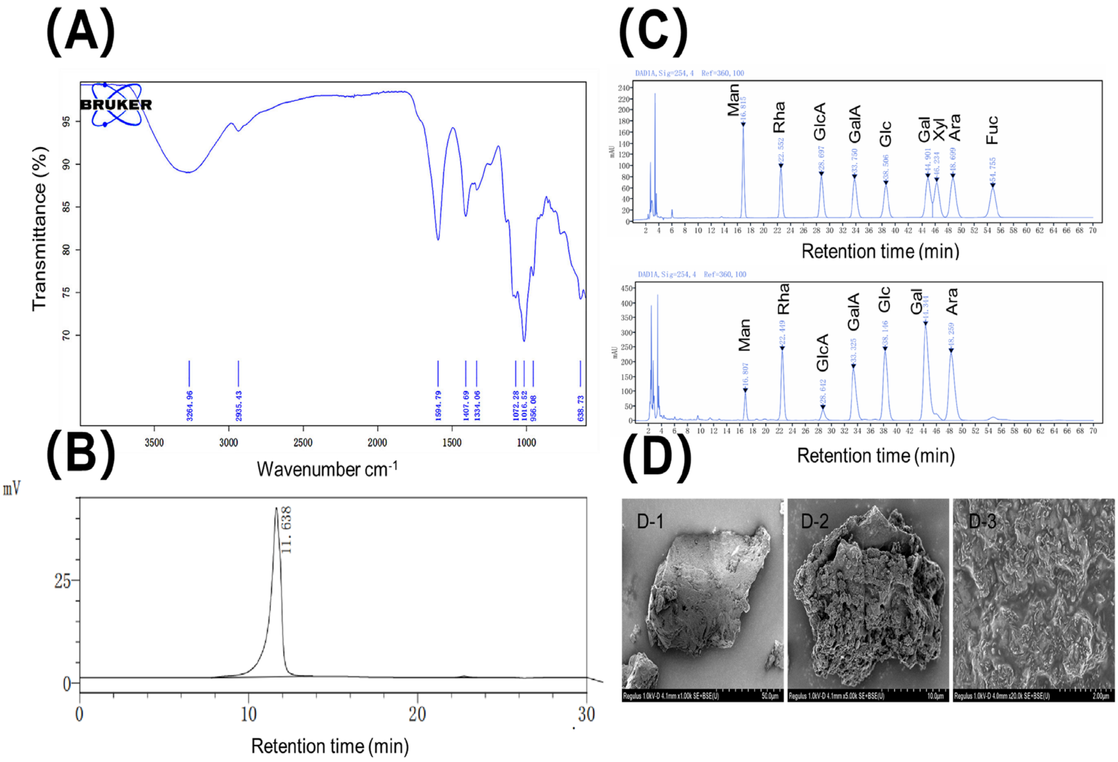

Figure S2.

(A) FT-IR spectrum of ASPS.

(B) High-performance gel permeation chromatography (HPGPC) spectrum of ASPS.

(C) The HPLC chromatograms of PMP derivatives.

Monosaccharide standards (upper panel); D-mannose (Man), D-rhamnose (Rha), D-glucuronic acid (GlcA), D-galacturonic acid (GalA), D-glucose (Glc), D-galactose (Gal), D-xylose (Xyl), L-arabinose (Ara), D-fucose (Fuc).

Monosaccharide composition of ASPS (lower panel); D-galactose (Gal), D-glucose (Glc), L-arabinose (Ara), D-rhamnose (Rha), D-galacturonic acid (GalA), D-mannose (Man), and D-glucuronic acid (GlcA) in the molar ratio of 11:9:7:5:5:1:1.

(D) SEM images of the ASPS (D-1:×1.0K; D-2:×5.0K; D-3:×20.0K).

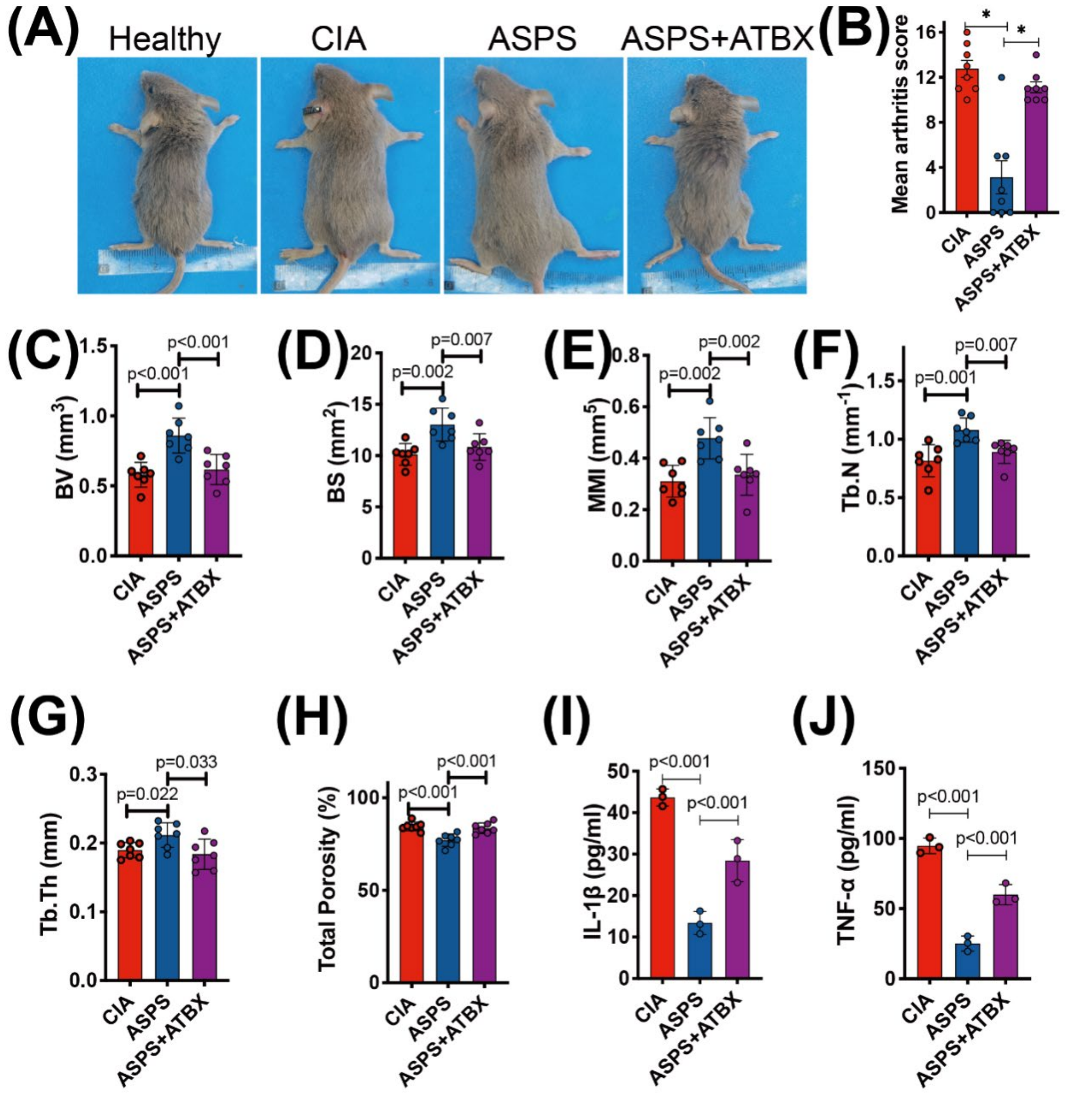

Figure S3.

(A) Representative images of mice on the 14th day after primary immunization.

(B) Clinical arthritis score of the mice on the 51st day after primary immunization. (n = 8)

(C-H) Quantitative analysis of bone volume (BV), bone surface (BS), polar moment of inertia (MMI), trabecular number (Tb. N), trabecular thickness (Tb. Th), total porosity. (n = 7)

(I-J) Serum samples were collected for measuring IL-1β and TNF-α release by ELISA.

Statistical analyses were performed with ANOVA followed by the post hoc Student-Newman-Keuls pairwise comparisons (C-J), or Kruskal-Wallis One Way Analysis of Variance on Ranks followed by the post hoc Tukey's comparisons (B). Error bars indicate SEM. *$p < 0.05$.

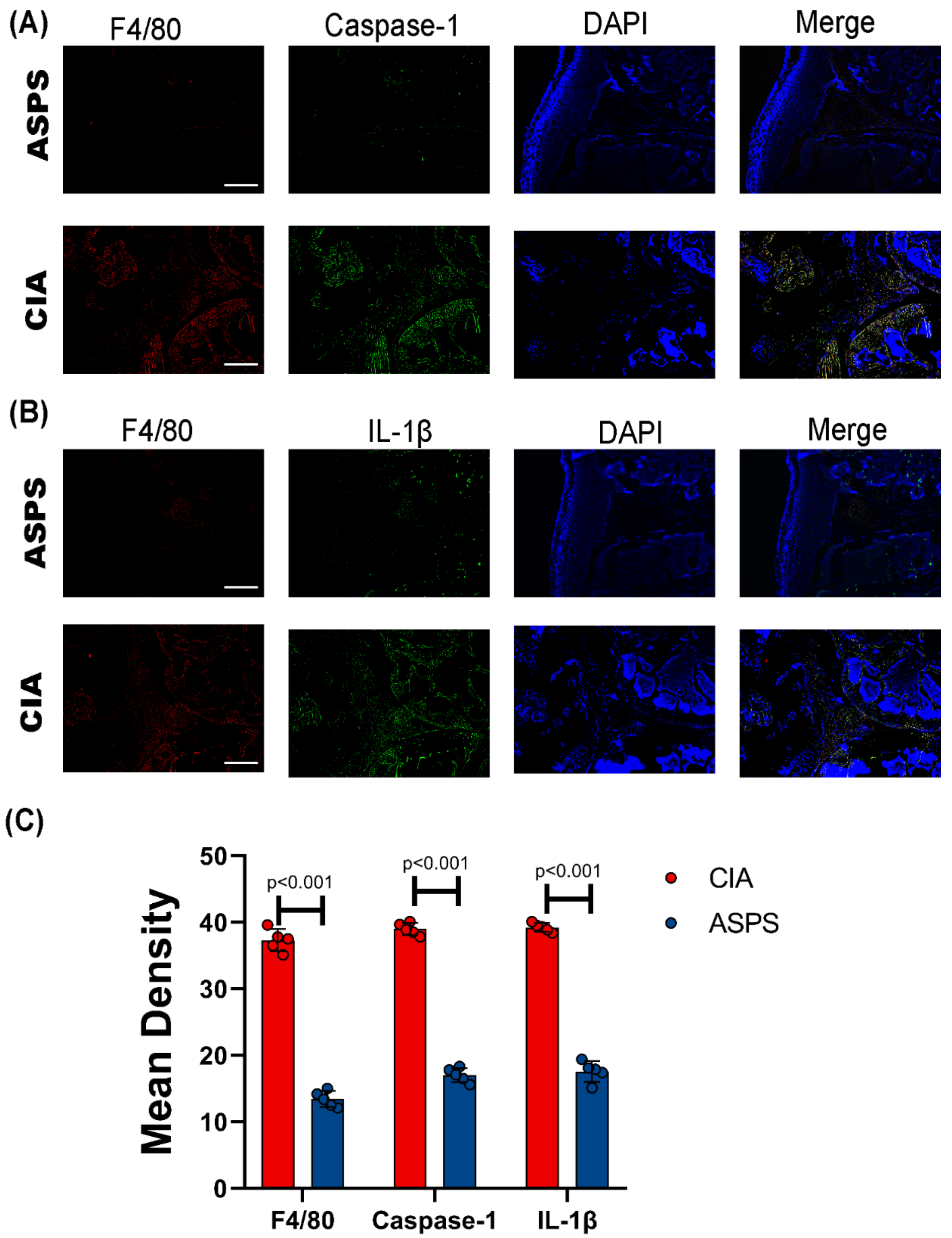

Figure S4. ASPS could inhibit inflammasome activation in macrophages in vivo. (A-B) Immunofluorescence staining of F4/80, Caspase-1 and IL-1β in joint specimens. Scale bar: 100 μm.

(C) Quantification of F4/80, Caspase-1 and IL-1β using mean density (integrated

density/specimen area) in joint specimens. n=5.

Statistical analyses were performed with ANOVA followed by the post hoc Student-Newman-Keuls pairwise comparisons (C). Error bars indicate SEM.

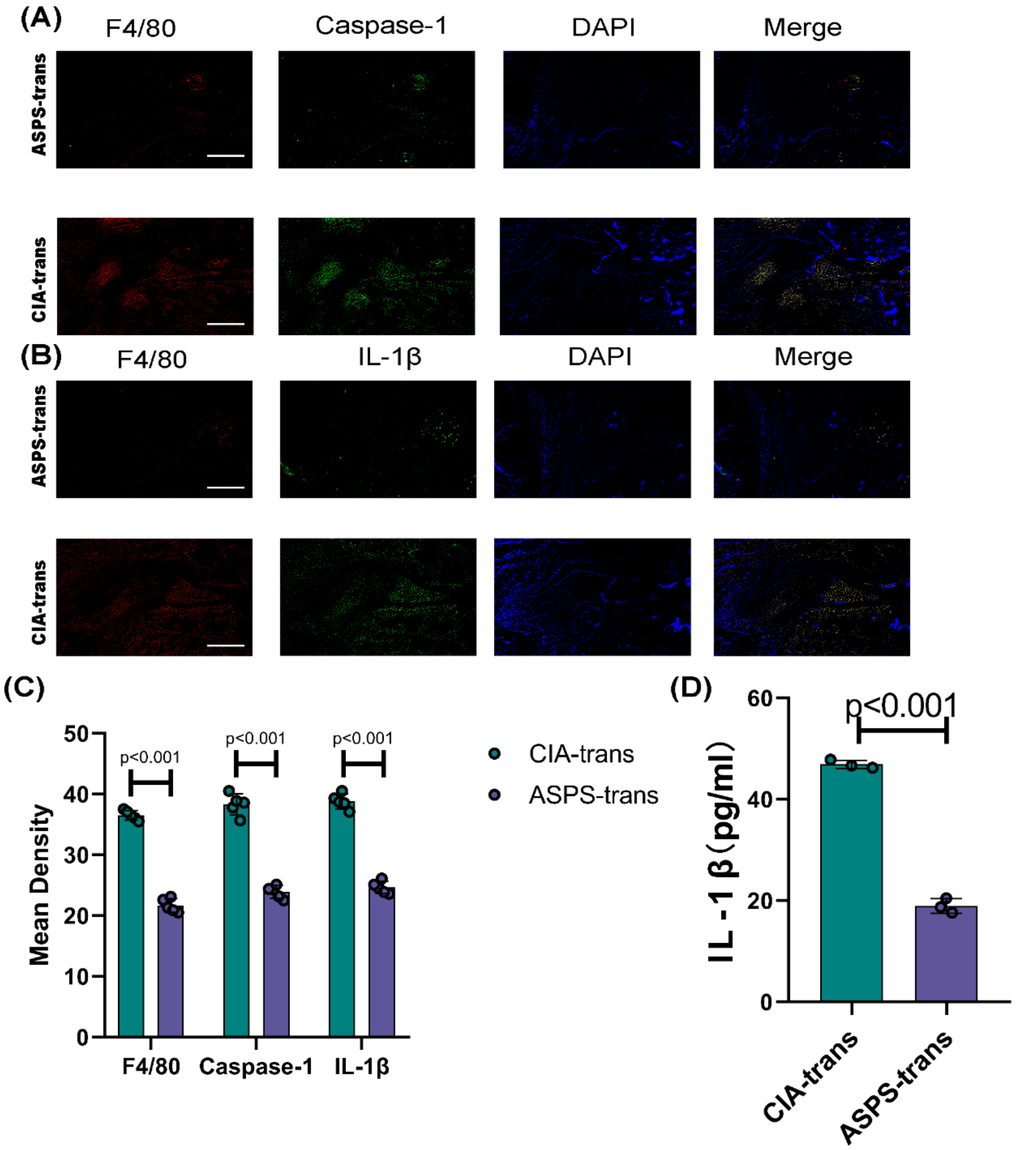

Figure S5. Fecal microbiota transplantation could inhibit inflammasome activation in macrophages in vivo.

(A-B) Immunofluorescence staining of F4/80, Caspase-1 and IL-1β in joint specimens. Scale bar: 100 μm.

(C) Quantification of F4/80, Caspase-1 and IL-1β using mean density (integrated density/specimen area) in joint specimens. n=5.

(D) Serum samples were collected for measuring IL-1β release by ELISA.

Statistical analyses were performed with ANOVA followed by the post hoc

Student-Newman-Keuls pairwise comparisons (C, D). Error bars indicate SEM.

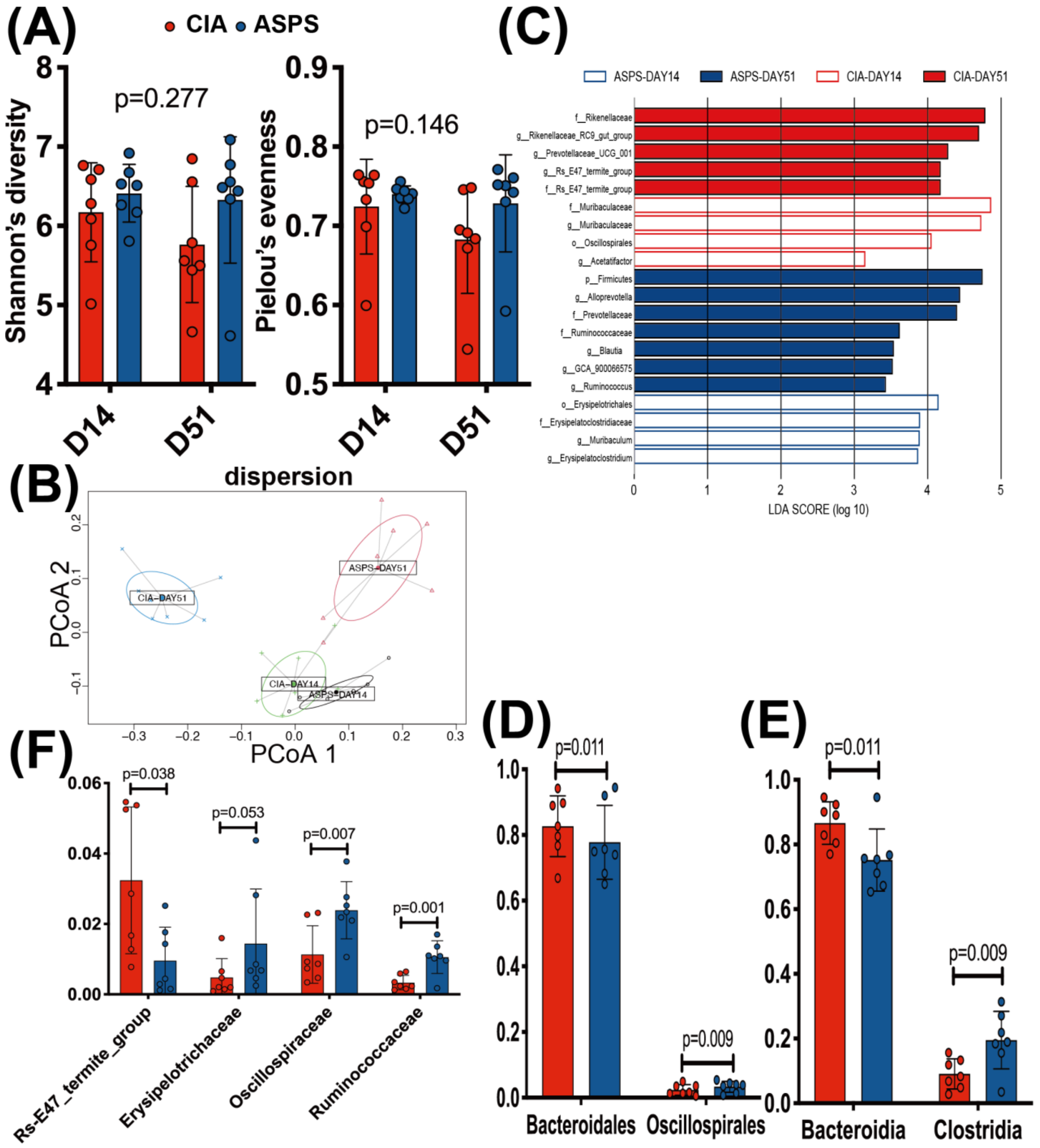

Figure S6.

(A) Alpha diversity indices of CIA and ASPS-treated mice on the 14th and 51st day after primary immunization, including the Shannon and Pielou's evenness indices. (n = 7)

(B) Bray-Curtis distance-based betadisper analysis. (n = 7)

(C) Change of gut microbiota in different phylogenetic levels, identified by LEfSe analysis of microbial samples with cutoff LDA = 3. (n = 7)

(D-F) Relative abundance of differential gut microbes identified by LEfSe analysis of microbial samples on the 51st day after primary immunization at class, order, and family level. (n = 7)

Statistical analyses were performed with ANOVA (A), or unpaired t-test (D-E, F [*Oscillospiraceae*, *Ruminococcaceae*]) or Mann-Whitney U-test (F [Rs-*E47_termite_group*, *Erysipelotrichaceae*]). Error bars indicate SEM.

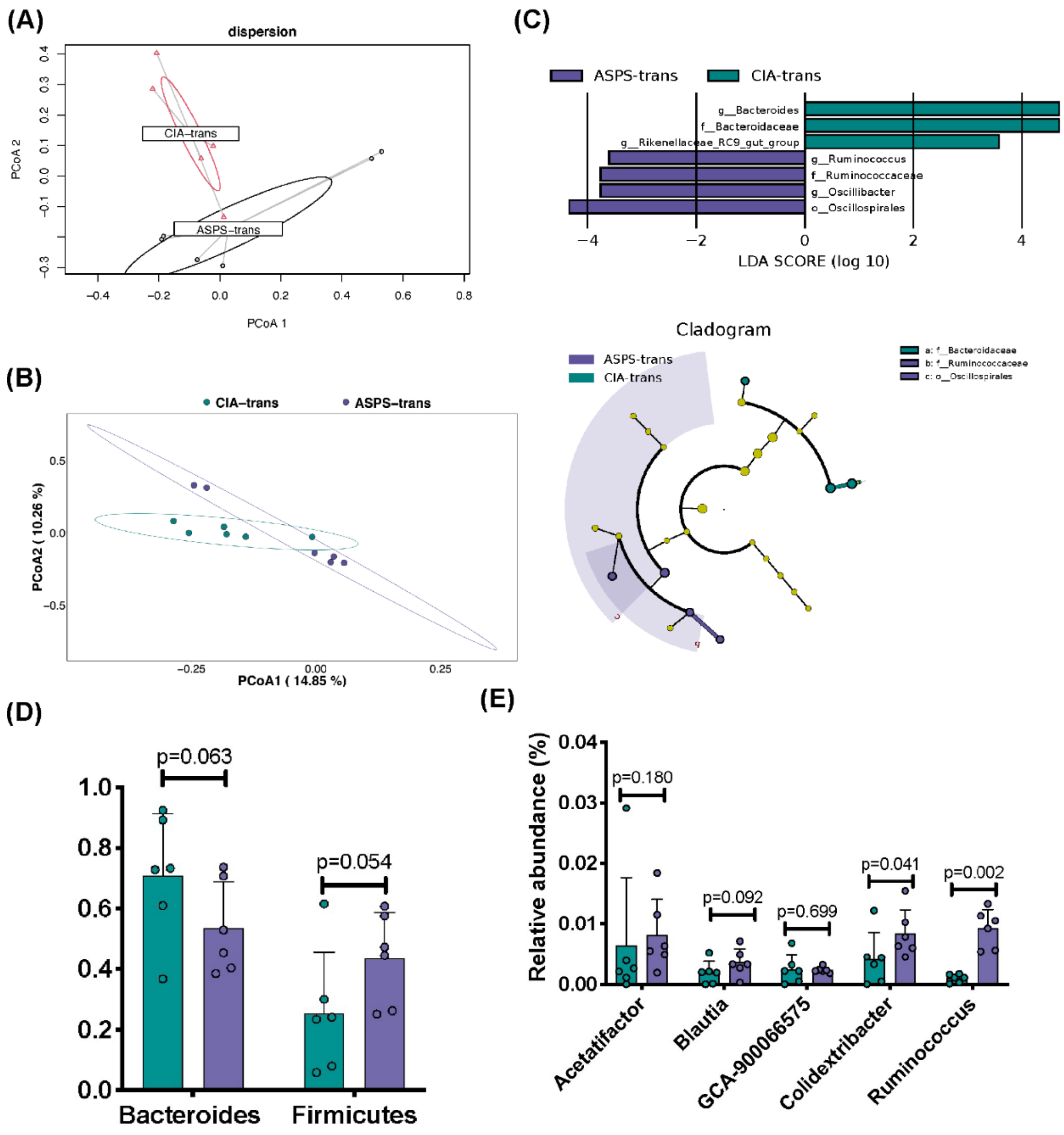

Figure S7. Fecal microbiota transplantation reprogrammed the gut microbiota of CIA mice.

(A) Bray-Curtis distance-based betadisper analysis. (n = 6)

(B) Bray-Curtis distance-based Principle Coordinate Analysis (PCoA) plot from mice in CIA-trans and ASPS-trans groups on the 51st day after primary immunization. (n = 6)

(C) LEfSe analysis of microbial samples on the 51st day after primary immunization with cutoff LDA = 3. (n = 6)

(D-E) Relative abundance of differential gut microbes identified by LEfSe analysis of microbial samples on the 51st day after primary immunization at phylum, and genus level. (n = 7)

Statistical analyses were performed with PERMANOVA (A), unpaired t-test (D), or Mann-Whitney U-test (E). Error bars indicate SEM.

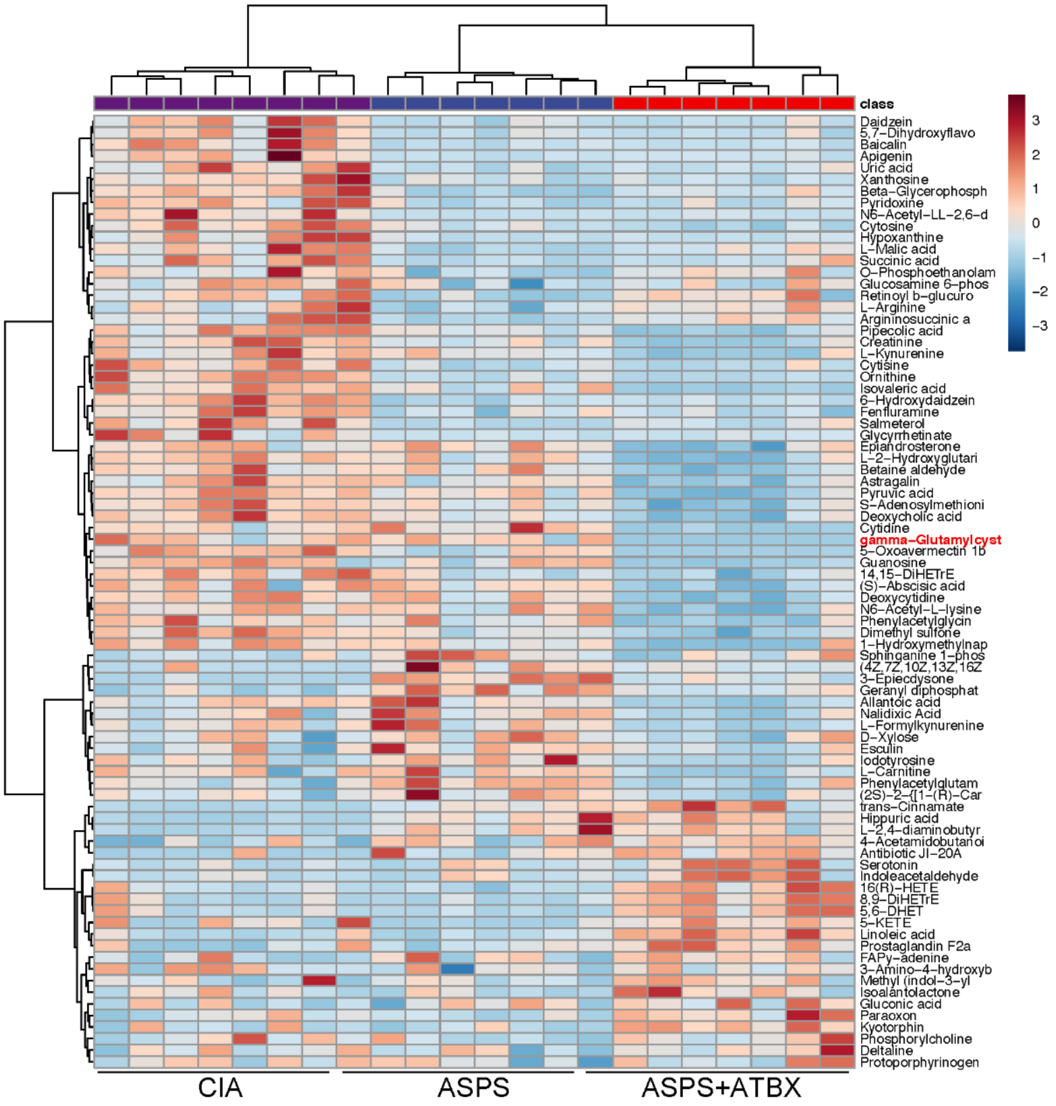

Figure S8.

Heatmap plot of differential serum metabolites, identified by one-way ANOVA with adjusted p-value (FDR) < 0.05

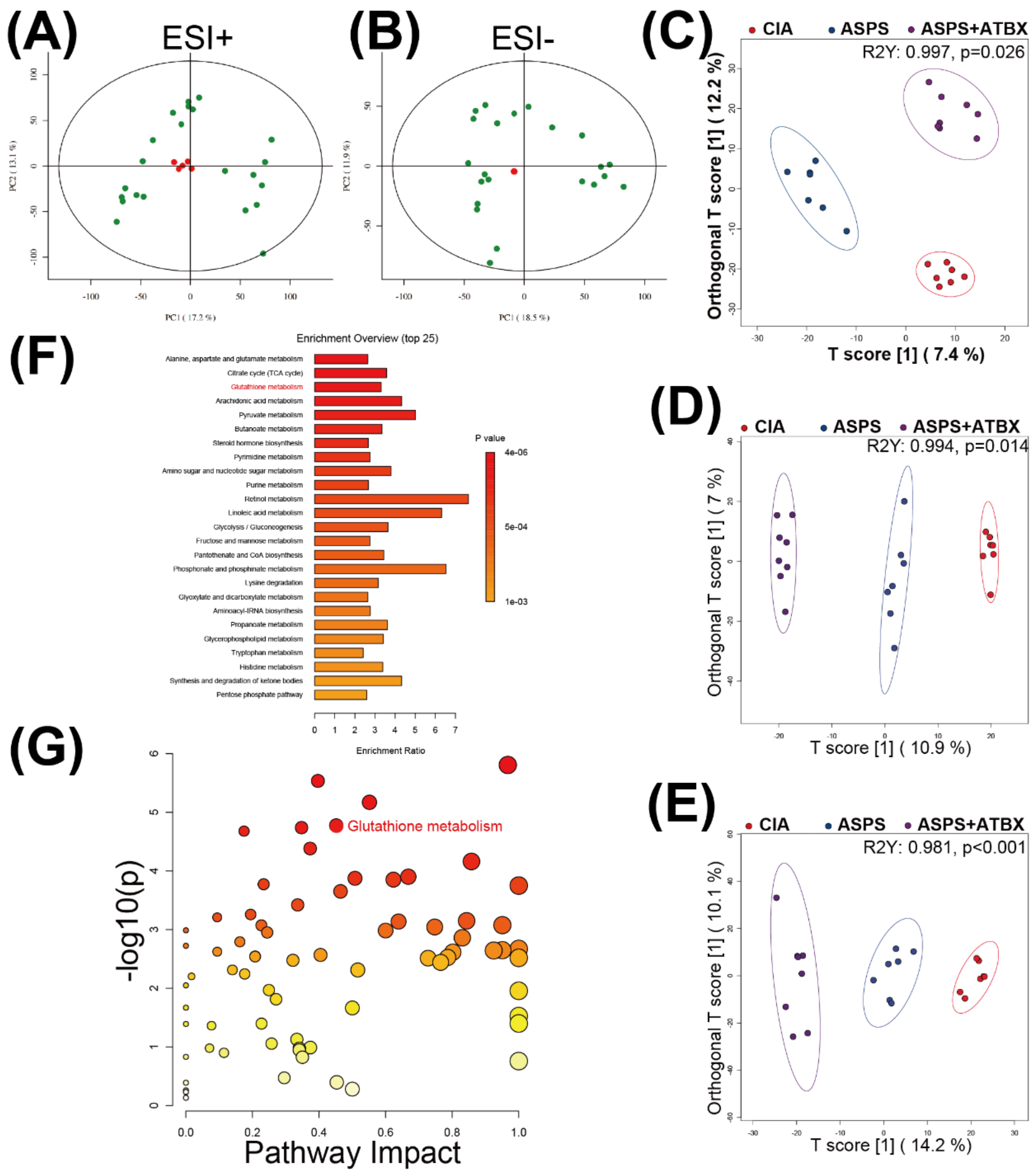

Figure S9.

(A-B) Principal component analysis (PCA) score plots of metabolomics data in ESI+/- modes. Red symbols represent the data of quality control samples.

(C-E) The Orthogonal Partial Least-squares discrimination analysis (OPLS-DA) of the serum metabolome (ESI+, ESI-, and MS1 features). Each symbol represents the data of an individual mouse. (n = 7 or 8)

(F-G) The enriched KEGG pathways of altered MS1/2 features.

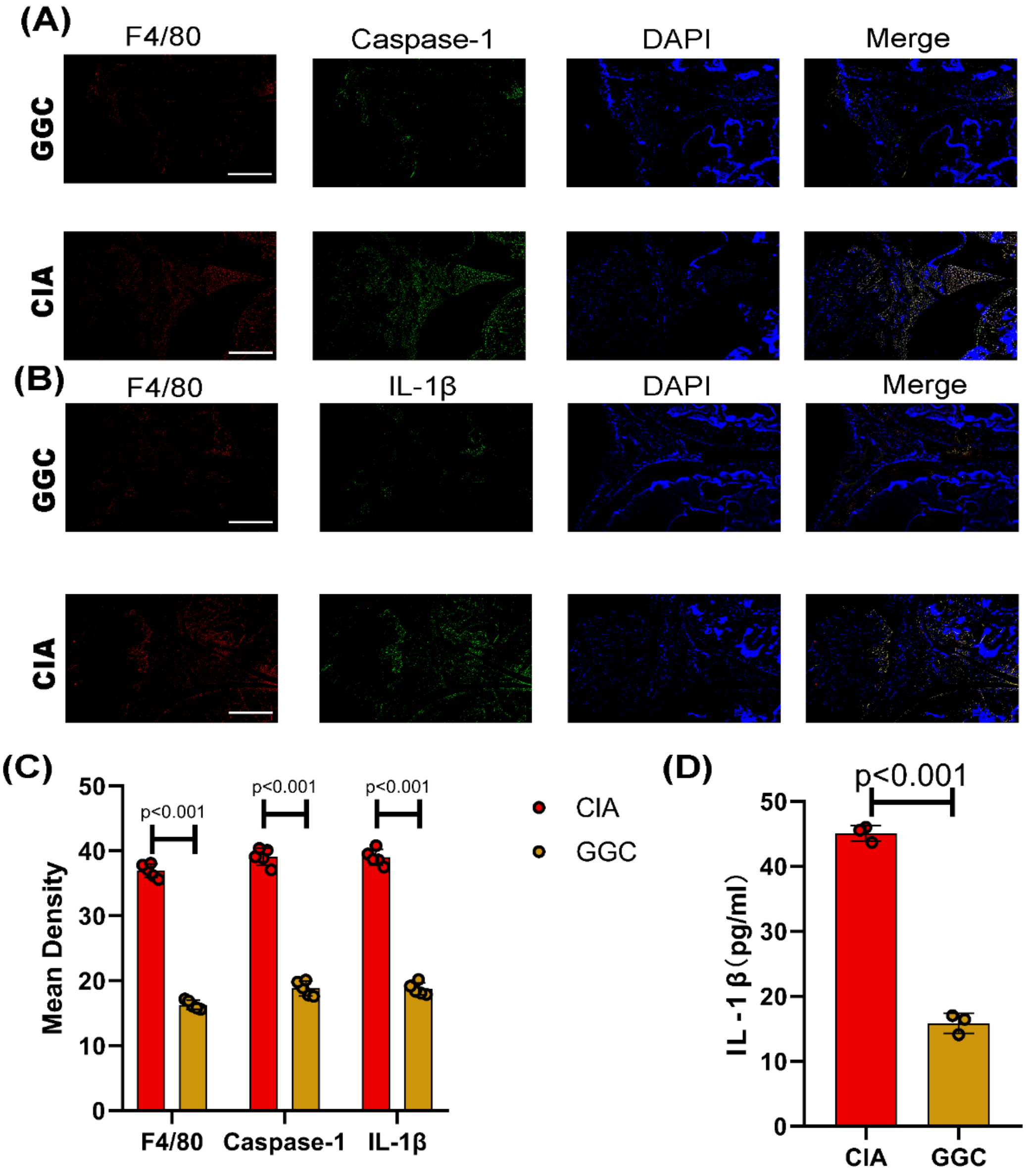

Figure S10. γ-glutamylcysteine could inhibit inflammasome activation in macrophages in vivo.

(A-B) Immunofluorescence staining of F4/80, Caspase-1 and IL-1β in joint specimens. Scale bar: 100 μm.

(C) Quantification of F4/80, Caspase-1 and IL-1β using mean density (integrated density/specimen area) in joint specimens. n=5.

(D) Serum samples were collected for measuring IL-1β release by ELISA.

Statistical analyses were performed with ANOVA followed by the post hoc

Student-Newman-Keuls pairwise comparisons (C, D). Error bars indicate SEM.

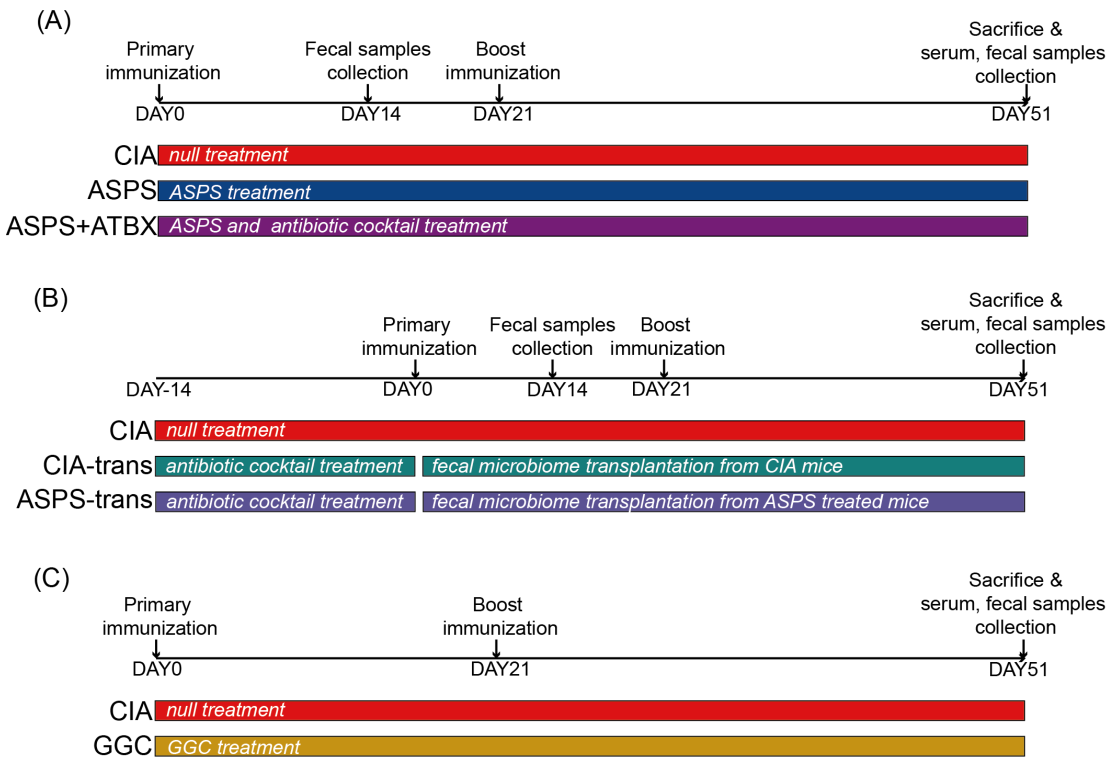

Figure S11.

(A) Outline of ASPS treatment experiment.

(B) Outline of fecal microbiota transplantation experiment.

(C) Outline of γ-glutamylcysteine (GGC) treatment experiment.

Table S1. List of the molecular docking research between GGC with the NLRP3, ASC, Caspase-1

Target protein	PDB ID	Binding energy (kcal/mol)	Binding residues of target protein with GGC
NLRP3	6NPY	-5.7	His520, Arg165, Trp414, Glu150
ASC	6KI0	-5.6	Glu45, Glu153, Arg344, Tyr341, Trp62, Arg66, Asp65
Caspase-1	2FQQ	-3.7	Glu390, Cys331, Arg391, Glu241, Asn259
